# Spatial associations between plants and vegetation community characteristics provide insights into the processes influencing plant rarity

**DOI:** 10.1371/journal.pone.0260215

**Published:** 2021-12-20

**Authors:** Meena S. Sritharan, Ben C. Scheele, Wade Blanchard, David B. Lindenmayer

**Affiliations:** 1 Threatened Species Recovery Hub, Fenner School of Environment and Society, The Australian National University, Canberra, Australian Capital Territory, Australia; 2 Fenner School of Environment and Society, The Australian National University, Canberra, Australian Capital Territory, Australia; Seoul National University, REPUBLIC OF KOREA

## Abstract

Determining the drivers of plant rarity is a major challenge in ecology. Analysing spatial associations between different plant species can provide an exploratory avenue for understanding the ecological drivers of plant rarity. Here, we examined the different types of spatial associations between rare and common plants to determine if they influence the occurrence patterns of rare species. We completed vegetation surveys at 86 sites in woodland, forest, and heath communities in south-east Australia. We also examined two different rarity measures to quantify how categorisation criteria affected our results. Rare species were more likely to have positive associations with both rare and common species across all three vegetation communities. However, common species had positive or negative associations with rare and other common species, depending on the vegetation community in which they occurred. Rare species were positively associated with species diversity in forest communities. In woodland communities, rare species were associated negatively with species diversity but positively associated with species evenness. Rare species with high habitat specificity were more clustered spatially than expected by chance. Efforts to understand the drivers of plant rarity should use rarity definitions that consider habitat specificity. Our findings suggest that examining spatial associations between plants can help understand the drivers of plant rarity.

## Introduction

Understanding where and why rare plants occur has long been a central question in ecology [1, 2]. An exploratory avenue to examine the ecological drivers of plant rarity involves analysing spatial associations of plant species in vegetation communities. Spatial associations in species co-occurrence data can be a potential sign of biotic interactions between plant species [3]. While species co-occurrence data is not a proxy for ecological interactions [4], evidence suggests that spatial associations can help understand the assembly and dynamics of plant communities [5–8].

Plant species can have direct positive and negative associations with each other [9–11]. Using spatial associations as a proxy for positive and negative associations between species can indicate how niche differentiation influences the occurrence of rare species [8]. Niche-coexistence theory predicts that species coexistence is due to differentiation in resource use [12, 13]. Positive spatial associations between species can indicate differentiation in resource use [14], enabling many rare species to persist within a community. Positive associations also can indicate non-competitive or facilitative interactions between species [15, 16]. In contrast, spatial segregation of species can suggest negative, competitive associations between species. Negative associations between species may inhibit the presence and abundance of rare species [17]. In resource-rich communities, competition can reduce niche space and availability [18], leading to a lower portion of rare species present within a community. The growth form of species also can influence the presence and abundance of rare species through positive or negative effects on species occurrence patterns. Therefore, examining the associations present between rare and common species within a vegetation community can help identify the occurrence patterns of rare plants.

Vegetation community characteristics and the spatial clustering patterns of rare species can indicate how niche availability may drive patterns of rare plant occurrence. Identifying relationships between the presence of rare species with vegetation community metrics can assist in understanding ecological processes that may influence plant rarity [19, 20]. The number of niches available in a local environment limits the number of species present [21]. Vegetation communities with high species diversity, evenness and high species density suggest high niche availability [22]. In such cases, many rare species can occupy different parts of the niche space and co-exist with limited competition due to dissimilarities in plant traits and resources required [14]. A vegetation community with low diversity and evenness (a system dominated by a single or a few species) may indicate fewer niches available to occupy and high competition for resources, leading to fewer rare plants [20]. Many rare species clustering within a local environment suggests that niche differentiation may influence the presence of rare species [23]. In environmentally stressful environments, plants tend to cluster together, profiting from the presence of other species [24–26]. Species clustering can result from facilitation between species [27] or the presence of microhabitats that support a high diversity of rare species [28].

Our study addressed the following questions: 1) Are positive and negative associations between plant species associated with the occurrence of rare plants, and 2) Are patterns of rare species clustering and vegetation community characteristics associated with the occurrence patterns of rare plants? To answer these questions, we completed vegetation surveys of 86 sites in woodland, forest, and heath communities in south-east Australia. We then examined the different types of associations present between rare and common plants and sought to determine if they differed across woodland, forest, and heath communities. As these vegetation communities differ in resource availability and disturbance [29], we predicted that: 1) The occurrence of rare plants will be correlated with positive associations between species [30, 31], and 2) rare plants will be more likely to be found in clusters [28] and in sites of high species diversity, evenness, and density [22, 32].

As there are different ways to define rarity [2, 33–35], we also examined whether different rarity measures influenced the relationships between plant rarity and the plant associations observed. Species rarity defined by low abundance [34] can indicate how competition can influence plant rarity [31], and changes in abundance can indicate if niche availability limits abundance. In contrast, definitions of rarity based on more than abundance, such as Rabinowitz’s [33] seven forms of rarity, may indicate how distribution and habitat preferences influence plant rarity. Applying different rarity types can help determine the drivers of rarity and vulnerability of species to environmental change [36].

## Methods

Permission to conduct vegetation surveys across Booderee National Park was given by Parks Australia (PA2018-00020).

### Study area

We conducted our study in Booderee National Park (BNP), a ~6,500-ha reserve located on a coastal peninsula in south-eastern Australia (35°40’ S, 150°40’ E). The region has a temperate maritime climate with an average annual rainfall of 1212 mm over the last 20 years [37]. During a five-month survey period between September 2018 and February 2019, the mean monthly rainfall was 85 mm, while the mean maximum temperature was 25.6°C and the mean minimum temperature was 15.8°C. BNP supports floristically diverse Sydney Coastal Heath and Sydney Coastal Dry Sclerophyll Forest vegetation communities [38, 39]. The park is dominated by dry sclerophyll vegetation consisting primarily of forest (45.1% of BNP) followed by heath (15.3%) and woodland (12.9%) vegetation communities [40].

The forest vegetation community (where the trees have touching crowns, >20m tall, [40]) is the most extensive vegetation community across BNP, dominated by *Eucalyptus pilularis*, *Corymbia gummifera*, and *Eucalyptus botryoides*. The mid-storey comprises *Banksia serrata*, *Acacia longifolia*, and *Monotoca eliptica*, while the understory is dominated by *Pteridium esculentum* and *Lomandra longifolia*.

Woodland communities (where the trees have separated crowns, <20m tall [40];) have high variation in crown cover, from closely spaced to widely separated, and are distinguished from forests based on lower stature and species composition. The overstorey is comprised of *Eucalyptus sclerophylla*, *C*. *gummifera*, *and B*. *serrata*; the mid-storey is dominated by *B*. *serrata* and *C*. *gummifera*, and the understory is composed of *P*. *esculentum*, *B*. *serrata*, *Lambertia formosa*, *Acacia longifolia*, *Acacia suaveolens*, and *L*. *longifolia*. Woodland vegetation communities occur in transitional areas between forests and heaths [40].

Heath communities are treeless and are dominated by a variety of shrub species usually less than two metres tall with small narrow leaves forming part of the canopy [40]. Dry heath communities are dominated by *Banksia ericifolia*, *Allocasuarina distyla*, *Sprengelia incarnata*, *Baeckea imbricata*, *Isopogon anemonifolius* and *Hakea teretifolia*, while in wet heath *Gahnia clarkei*, *Gleichenia dicarpa*, *Leptospermum* or *Melaleuca* species also can be found.

### Field methods

We surveyed long-term monitoring sites first established in 2003 to assess biodiversity responses to fire [41] (all surveys completed by M.S to ensure consistency in survey protocols and plant identification). In our study, we surveyed 86 sites which covered the three major vegetation communities in BNP: forest (39 sites), woodland (22 sites) and heath (25 sites). We selected our sites to ensure that the number of sites per vegetation community was in proportion to the area the vegetation community covered.

We established a 10 m by 10 m plot at each of the 86 sites and surveyed 100 one metre by one-metre quadrats within the plot by creating a ten by ten-unit grid. We identified and recorded the presence of all plant species within each one metre square quadrat. If an individual plant had its stem or foliage cover across more than one quadrat, its location was assigned to the quadrat where the greatest part of the stem of the plant was located. We identified each plant to species level. If plants could not be identified to the species level, they were given a unique identifier.

We recorded the total abundance of a species at a site as the number of one-metre quadrats in which a species was present. We counted species in relation to their presence instead of the cover of a species within each one metre by one metre quadrat. Plant cover was difficult to assess accurately in vegetation-dense sites due to overlapping vegetation greater than two metres in height. We acknowledge that if small species were highly abundant in a quadrat, but not across multiple quadrats in a site, they could have been classified as rare, despite potentially being locally abundant in a proportion of a sites. However, this sampling method does not affect our data analysis as we sought to effectively detect changes in the distribution and abundance of species populations across sites [42]. We conducted plant surveys from September 2018 to February 2019, mostly during the prime flowering season. For the full list of identified species, see Table 1 in S1 Appendix.

### Plant rarity classification

We used two methods that have been employed widely in the literature to categorise rarity. First, we used Gaston’s [34] measure of rarity by abundance by classifying species that occurred in the lowest 25^th^ quantile of species abundance as rare and those higher than the 25^th^ quantile as common. Second, we used Rabinowitz’s [33] seven forms of rarity (Table 1) to classify a species as rare, determined by having a low abundance, a narrow distribution and high habitat specificity. We calculated species abundance by averaging the abundance for each species across all sites and all vegetation communities. We determined the distribution of a species as the number of different sites where a species was found. We determined the habitat specificity of a species by quantifying how many vegetation communities of the three studied vegetation communities in which it was found. Plants with low habitat specificity were found across all three vegetation communities, whereas plants with high habitat specificity were found in only one vegetation community. Species classified as having a small local population size, narrow distribution, and high habitat specificity (NSS; Table 1) were considered to be the rarest under the seven forms of rarity categorisation and thus classified as rare in our study.

**Table 1:** Rabinowitz’s (1981) categorical classification of the seven forms of rarity for species, based on species abundance, distribution and habitat specificity.

	Rabinowitz definitions			Definitions applied to the study area		
Rarity category	Distribution	Population size	Habitat specificity	Distribution (number of sites)	Average abundance across all sites	Habitat specificity
WLU (common)	wide geographic distribution	somewhere large	unspecific	> 40 sites	> 50%	3
WSU	wide geographic distribution	everywhere small	unspecific	> 40 sites	< 50%	3
WLS	wide geographic distribution	somewhere large	specific	> 40 sites	> 50%	1
WSS	wide geographic distribution	everywhere small	specific	> 40 sites	< 50%	1
NLU	narrow geographic distribution	somewhere large	unspecific	< 40 sites	> 50%	3
NSU	narrow geographic distribution	everywhere small	unspecific	< 40 sites	< 50%	3
NLS	narrow geographic distribution	somewhere large	specific	< 40 sites	> 50%	1
NSS (rare species)	narrow geographic distribution	everywhere small	specific	< 10 sites	< 50%	1

### Data analyses

We repeated the analyses described below for species categorised using the two different definitions of rarity. For analyses involving linear models, species that were not identified as rare with Rabinowitz’s rarity measure were classified as ’not rare’. For all analyses, we included all 455 presumed species found. However, 67 of these species were not identifiable (15.6%).

#### We conducted our analyses in R version 4.0.2 [43]. The full dataset is available at doi: 10.5061/dryad.2z34tmpnp positive and negative associations between species

To determine if rare species were positively or negatively associated with other rare and common species across a vegetation community at a landscape scale, we used the method developed by Calatayud et al. [31]. We chose to examine the spatial associations between species within each vegetation community. Spatial associations at this scale enabled us to examine how species may be associated across different sites, as there were insufficient numbers of each rare species at the plot level to detect significant positive or negative associations between species. Following Calatayud et al. [31], we calculated the similarity in abundance distribution across all sites within forest, woodland and heath vegetation communities for each species pair *i* and *j* using Schoener’s index [44]. We compared the observed similarities to 999 null values we obtained through the randomisation of species abundances using a fixed-fixed null model. This null model randomises species abundances while keeping constant marginal totals of the rows and columns of the species abundance matrix. We used the "r2dtable" algorithm from the "vegan" package [45] for the null model. For each observed similarity value, we calculated two one-tailed p-values as the proportion of null values (plus the observation) that were higher than or equal to the observed value to identify a positive association. To identify a negative association between species, we calculated two one-tailed values for each observed similarity value as the proportion of null values (plus the observation) that were lower than or equal to the observed value. We considered an association significant when the associated p-values in any of the two tests were lower than the 0.05 probability threshold.

With the outputs derived using Calatayud et al. [31] ’s function, we first ran a negative binomial generalised linear model to examine if the number of positive associations a species had was related to the growth form and the rarity of a species within each vegetation community. The number of positive associations a species had was the response variable, and the linear predictor for the model was: growth form * rarity. We then ran a model with growth form and rarity as an additive effect (growth form + rarity) and a third model that looked for an association between the number of positive associations a species had and the rarity of the species. We compared the AIC values for all three models, and we chose the model with the lowest AIC value. We then repeated the three models above, changing the response variable to the number of negative associations a species had within a community. We fit negative binomial generalised linear models using the "glm.nb" function in the "MASS" package [46].

We conducted two separate sensitivity analyses to ensure the positive and negative associations we observed were robust. To confirm whether the observed patterns of positive and negative associations were robust to a different probability threshold, we established positive or negative links based on a 0.01 probability threshold. This threshold provided similar results (S2 Appendix). We then explored whether an alternative null model might yield different results. We used the "quasiswap count" algorithm in the vegan package [45], which maintains zero entries in the species per sample matrix. We conducted the alternative null model with both a 0.05 and 0.01 probability threshold. The results remained mostly constant when using the alternative null model (S2 Appendix).

#### Rare species clustering

To determine if rare species were clustered within a site at a local scale, we used Moran’s I [47, 48], which quantifies levels of spatial dependence for each species. Our null hypothesis assumed there was no spatial dependence between rare species at a site. We used the row and column number of each quadrat at a site as coordinates. We generated a matrix of inverse distance weights where each off-diagonal entry (point i, point j) in the matrix was equal to 1/(distance between point i and point j). We calculated Moran’s I using the package ’ape’ [49] based on the presence of a species in each one-metre by one-metre quadrat within the 10 by 10-metre plot at each site. We did not consider sites where no rare species were identified (one site).

#### Associations between plant rarity and vegetation community metrics

We examined associations between three different vegetation community metrics with the number of rare species present at a site. Our vegetation community metrics were species diversity, density, and evenness. We quantified Shannon’s index of alpha diversity at a site using the ’vegetarian’ package [50]. We calculated species evenness using the ’vegan’ package [45]. We calculated species density as the total abundance of all species at a site. We used negative binomial generalised linear models using the "glm.nb" function in the "MASS" package [46], where the number of rare species present at a site was our response variable. For full model outputs see S3 Appendix.

## Results

Overall, we found 455 plant species across our surveyed sites. With Gaston’s measure of rarity, 231 species were considered rare, that is species that occurred in the lowest 25^th^ quantile of species abundance, while 224 species were classified as common. Using Rabinowitz’s measure of rarity, 262 species were classified as ’most rare’ (NSS), while four species were classified as the most common within our dataset (WSU); *Lomandra longifolia*, *Imperata cylindrica*, *Entolasia marginata*, and *Pteridium esculentum*.

In the forest community, 87 species were classified as rare and 105 species as common with Gaston’s measure of rarity. While 88 species were classified as ’most rare’ with Rabinowitz’s measure of rarity, 104 species were classified as ’not rare’; that is, they fell under one of the other seven categories of rarity (Table 1). In the woodland community, 120 species were rare, and 130 species were common under Gaston’s measure. With Rabinowitz’s measure, there were 81 rare species and 169 species classified as not rare. Across the heath community, 105 species were classified as rare and 136 species were classified as common under Gaston’s measure of rarity. Ninety-two species were classified as rare, and 149 species were classified as not rare with Rabinowitz’s measure of rarity.

Sixty-five of the 455 species were unidentifiable to the species level, with 55 of these species were classed as rare and ten were common under Gaston’s measure of rarity. Using Rabinowitz’s measure, all 65 unidentified species were considered ’most rare’. We were able to classify 57 species of the unidentifiable species to Genus level. Forty-three of the 57 species were classified as rare and 14 as common under Gaston’s measure of rarity. With Rabinowitz’s measure, 27 species were classified as ’most rare’ while the remaining 30 were not classified as rare.

### Positive and negative associations between plants

#### Gaston’s measure of rarity

**Fig 1:**
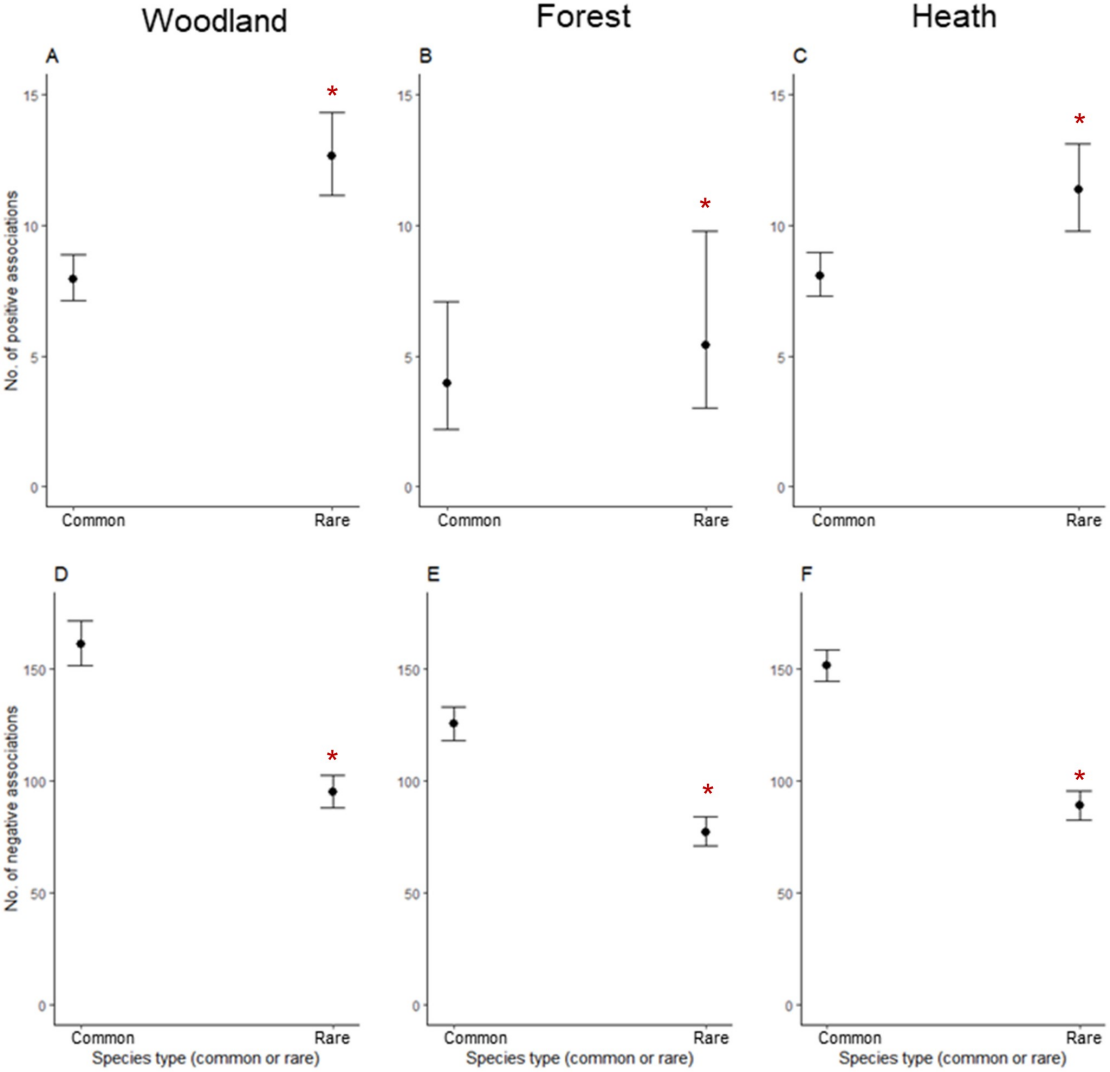
The number of positive and negative associations between species using Gaston’s rarity measure. The top row illustrates the number of positive associations for rare and common species in (A) woodland, (B) forest and (C) heath communities. The bottom row illustrates the number of negative associations both rare and common species had in (D) woodland, (E) forest and (F) heath communities. Asterisks (*) indicate significance at p < 0.05.

Rare plants had significantly more positive associations with all other plants than common species across woodland, forest, and heath communities (P < 0.01, Fig 1, Table 2). Rare species also had significantly fewer negative associations with all other plants across all three vegetation communities relative to common species (P < 0.001, Fig 1, Table 2). Species growth form did not influence the associations both rare and common species had across any of the three communities.

**Table 2:** Model outputs for the associations for species categorized as rare using Gaston’s classification had with other species using the ‘r2dtable’ algorithm and a 0.05 probability threshold.

Vegetation community	Woodland						Heath						Forest					
Type of association	No. of positive associations			No. of negative associations			No. of positive associations			No. of negative associations			No. of positive associations			No. of negative associations		
*Predictors*	*Log-Mean*	*CI*	*p*	*Log-Mean*	*CI*	*p*	*Log-Mean*	*CI*	*p*	*Log-Mean*	*CI*	*p*	*Log-Mean*	*CI*	*p*	*Log-Mean*	*CI*	*p*
(Intercept)	2.07	1.96–2.18	**<0.001**	5.08	5.02–5.14	**<0.001**	2.09	1.98–2.19	**<0.001**	2.09	1.98–2.19	**<0.001**	1.37	0.80–1.94	**<0.001**	4.83	4.77–4.89	**<0.001**
Rare Gaston	0.45	0.28–0.62	**<0.001**	-0.53	-0.63–0.43	**<0.001**	0.34	0.15–0.52	**<0.001**	0.34	0.15–0.52	**<0.001**	0.32	0.10–0.54	**0.004**	-0.48	-0.59–0.38	**<0.001**
Growth Form [graminoid]													-0.23	-0.87–0.42	0.489			
Growth Form [herb]													0.12	-0.47–0.70	0.696			
Growth Form [shrub]													0.14	-0.44–0.73	0.634			
Growth Form [tree]													-0.50	-1.21–0.21	0.166			
Observations	198			198			181			181			147			147		

Negative binomial generalised linear model outputs tested for the association between rarity of a species, classified with Gaston’s measure of rarity, and the number of positive and negative associations the species had with all other species. A positive association or negative association was considered significant where the associated p-values in any of the two tests were lower than the *0*.*05* probability threshold. Raw estimates, standardised regression coefficients and estimated 95% confidence intervals for the negative binomial generalised linear models are shown. The reference level (intercept) are species considered common by Gaston’s measure of rarity. In the model examining the number of positive associations in forest communities with rarity, the reference level (intercept) is the growth form, fern. Values in bold indicate significance at p < 0.05.

#### Rabinowitz’s measure of rarity

**Fig 2:**
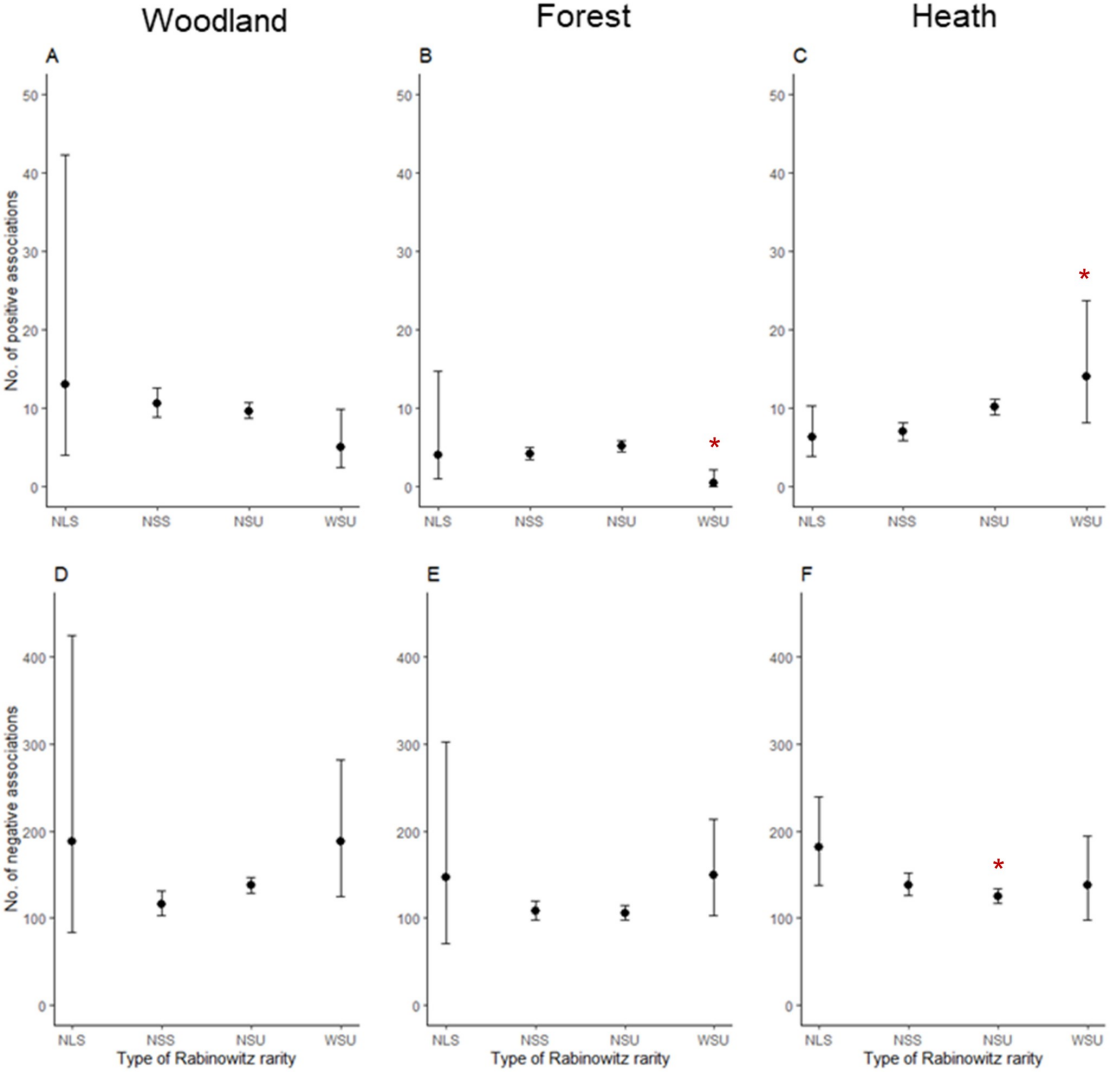
The number of positive and negative associations between species using Rabinowitz’s measure of rarity. Rabinowitz’s (1981) measure of rarity was classified according to the seven forms of rarity matrix, where species classified as having a small local population size, narrow distribution, and high habitat specificity (NSS) were considered most rare in our study. The top row illustrates the number of positive associations both rare and ‘not -rare’ species had (A) woodland, (B) forest and (C) heath communities. The bottom row illustrates the number of negative associations both rare and ‘not -rare’ had in (D) woodland, (E) forest and (F) heath communities. Asterisks (*) indicate significance at p < 0.05.

Across all three vegetation communities, species classified as most rare by Rabinowitz (NSS) did not have any significant positive or negative associations with all other plants. However, in the heath community, plants classified as having a narrow distribution, small population size, and low habitat specificity (NSU) had significantly fewer negative associations with other species (P = 0.012, Fig 2, Table 3). Plants classified as having a wide distribution, small population size, and low habitat specificity in heath sites (WSU; species considered most common) had a significantly high number of positive associations with other species (P = 0.026, Fig 2C, Table 3). In the forest community, WSU plants had significantly fewer positive associations with other species (P = 0.004, Fig 2B, Table 3). A species’ growth form did not influence the associations a species had, regardless of their Rabinowitz rarity categorisation across all three communities.

**Table 3:** Model outputs for the associations a Rabinowitz rare species had with other species using ‘r2dtable’ algorithm and a 0.05 probability threshold.

Vegetation community	Woodland						Heath						Forest					
Type of association	No. of positive associations			No. of negative associations			No. of positive associations			No. of negative associations			No. of positive associations			No. of negative associations		
*Predictors*	*Log-Mean*	*CI*	*p*	*Log-Mean*	*CI*	*p*	*Log-Mean*	*CI*	*p*	*Log-Mean*	*CI*	*p*	*Log-Mean*	*CI*	*p*	*Log-Mean*	*CI*	*p*
(Intercept)	2.56	1.48–3.93	**<0.001**	5.24	4.51–6.17	**<0.001**	1.85	1.36–2.35	**<0.001**	5.20	4.93–5.49	**<0.001**	1.60	1.01–2.20	**<0.001**	4.99	4.34–5.80	**<0.001**
Rare Rabinowitz [NSS]	-0.20	-1.58–0.90	0.740	-0.48	-1.43–0.26	0.253	0.10	-0.43–0.61	0.718	-0.27	-0.58–0.01	0.068				-0.30	-1.12–0.36	0.413
Rare Rabinowitz [NSU]	-0.30	-1.67–0.79	0.626	-0.31	-1.25–0.41	0.452	0.47	-0.04–0.97	0.067	-0.37	-0.66 –-0.09	**0.012**	0.18	-0.04–0.40	0.113	-0.33	-1.14–0.33	0.375
Rare Rabinowitz [WSU]	-0.96	-2.47–0.34	0.172	-0.00	-1.01–0.85	0.995	0.78	0.06–1.51	**0.035**	-0.27	-0.70–0.18	0.234	-2.11	-3.96 –-0.85	**0.005**	0.01	-0.86–0.77	0.974
Growth Form [graminoid]													-0.32	-0.98–0.34	0.342			
Growth Form [herb]													-0.09	-0.70–0.51	0.769			
Growth Form [shrub]													-0.06	-0.67–0.55	0.845			
Growth Form [tree]													-0.60	-1.32–0.12	0.105			
Observations	198			198			181			181			146			147		

Negative binomial generalised linear model outputs tested for the association between rarity of a species, classified with Rabinowitz’s measure of rarity, and the number of positive and negative associations the species had with all other species. A positive association or negative association was considered significant where the associated p-values in any of the two tests were lower than the *0*.*05* probability threshold. Raw estimates, standardised regression coefficients and estimated 95% confidence intervals are shown. The reference level (intercept) are species classified as NLS by Rabinowitz’s measure of rarity. In the model examining the number of positive associations in forest communities with rarity, the reference level is (intercept) is the growth form, fern. Values in bold indicate significance at p < 0.05.

### Rare species clustering

**Fig 3:**
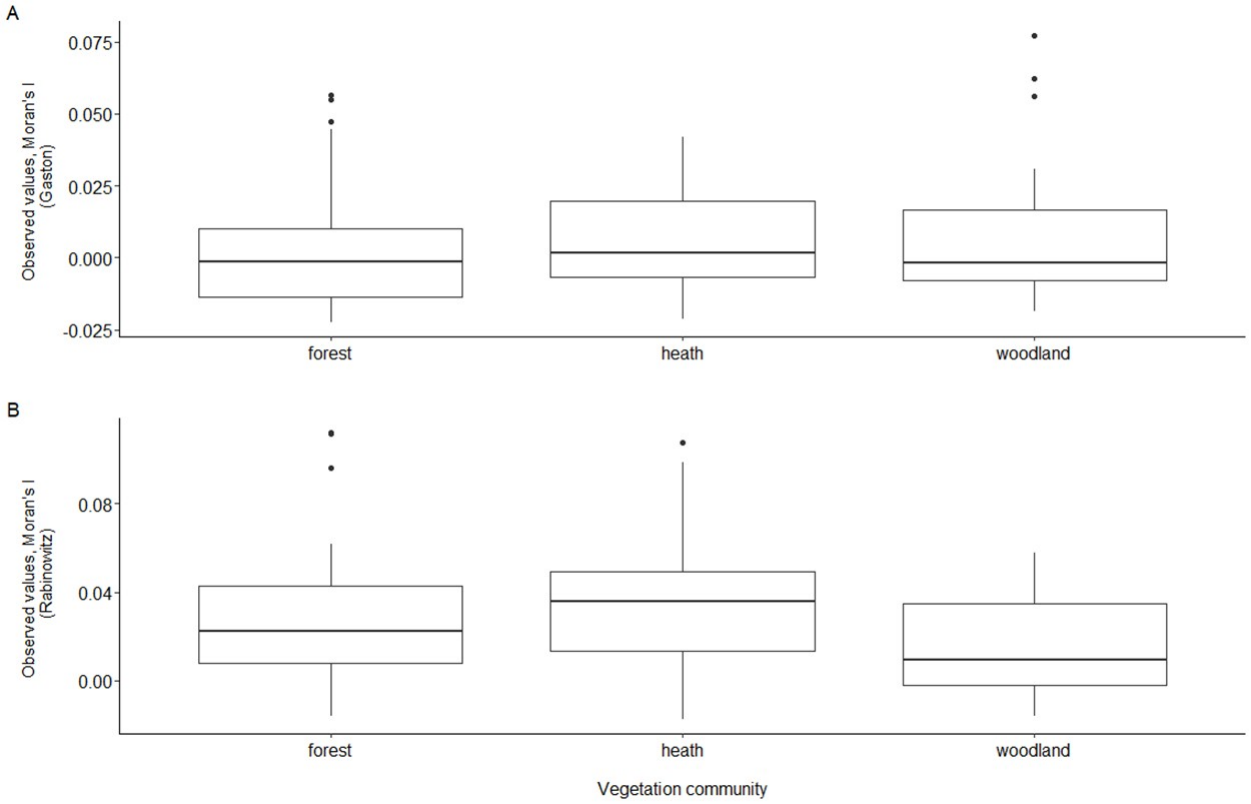
Observed values of Moran’s I for species clustering in sites across forest, heath, and woodland communities. (A) Site-level clustering of species classified as rare using Gaston’s measure of rarity. (B) Site-level clustering of species classified as rare through Rabinowitz’s classification system.

At the site level, rare species classified by Gaston’s measure of rarity were more clustered spatially than expected by chance for 27 of the 84 sites (31% of sites, p < 0.05). Species classified as rare using the Rabinowitz classification system were more clustered spatially than expected by chance alone on 50 of the 84 sites (59% of sites, p < 0.05). A linear model using the observed values of Moran’s’ I for each site showed that the site-level spatial clustering of species did not differ across all three vegetation communities for both measures of rarity (Fig 3).

### Associations between vegetation community characteristics and rarity

#### Gaston’s measure of rarity

**Fig 4:**
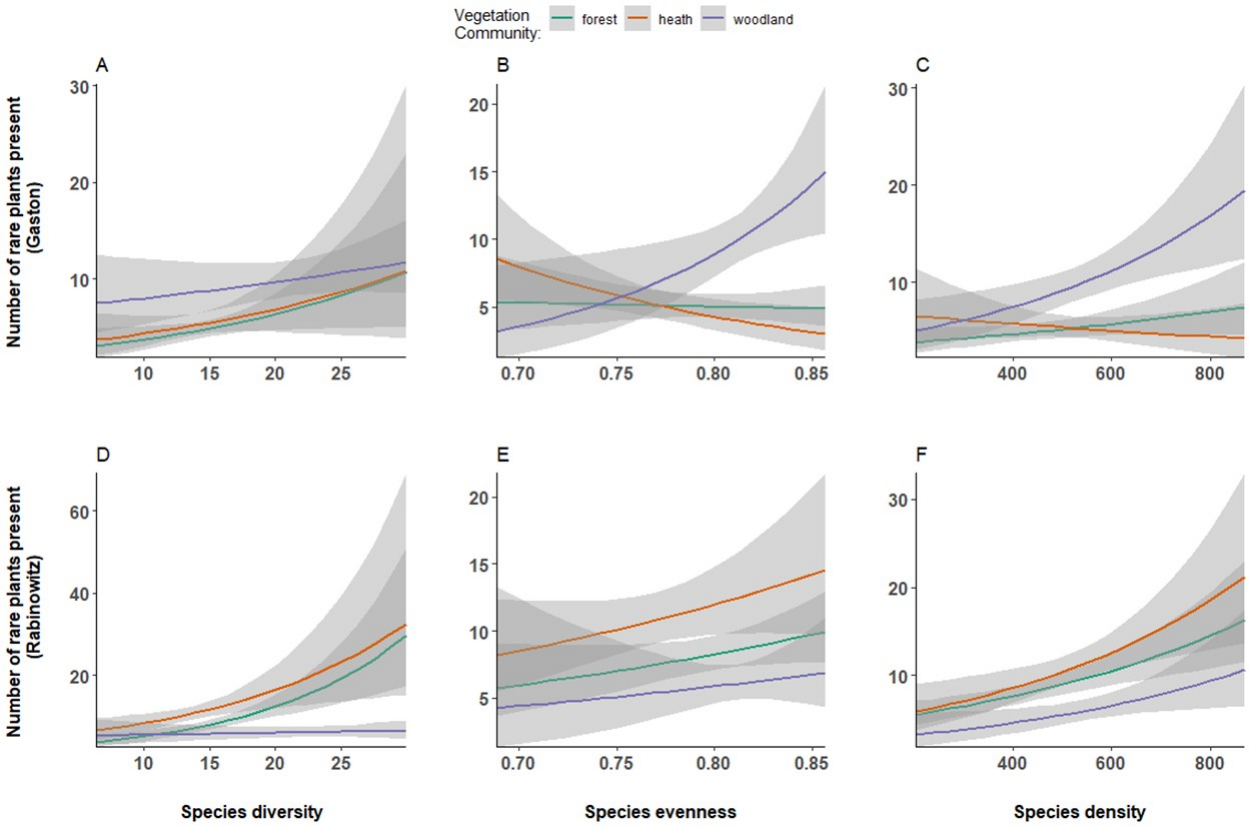
The number of rare species present in relation to species diversity, evenness, and density. The number of rare species (as classified under the Gaston system) present in relation to (A) species diversity, (B) species evenness and (C) species density at a site across forest (green), heath (orange) and woodland (purple) vegetation communities. The number of rare species classified using the Rabinowitz system present in relation to (D) species diversity, (E) species evenness and (F) species density at a site across all three vegetation communities.

The number of rare species in forest sites was positively associated with species diversity (P = 0.035, Fig 4A) but not in woodland and heath sites. The number of rare species present was significantly positively associated with species evenness in woodland communities (P = 0.025, Fig 4B) but not in heath or forest communities. Species density was not associated with the number of rare species present across all vegetation communities.

#### Rabinowitz’s measure of rarity

The number of rare species was associated negatively with species diversity in woodland communities (P = 0.001, Fig 4D). However, in forest communities, species diversity was positively associated with the number of rare species present (P< 0.001, Fig 4D). No association was present between the number of rare species in heath communities with species diversity. Species evenness was not associated with the number of rare species present at a site across all three communities. Species density was associated positively with the number of rare species present in forest communities (P < 0.001, Fig 4F), but there were no associations in woodland and heath communities.

## Discussion

Our study examined whether spatial associations, spatial clustering of rare species, and vegetation community metrics provide insights into processes shaping plant rarity patterns. We found that, based on Gaston’s measure of rarity, rare species were more likely to have positive associations with all other species. However, using Rabinowitz’s measure of rarity, species with different rarity classifications had vegetation community-specific positive and negative associations. Rare plants were more spatially clustered at a site than expected when using Rabinowitz’s rarity measure compared to Gaston’s measure of rarity. Plant rarity also was associated with species diversity, evenness and density in woodland and forest communities but not heath communities. We hypothesise that both plant rarity and commonness may, in part, be shaped by the positive and negative associations between species, driven by niche availability and competition for niche space.

### Positive and negative associations with plant rarity

Using Gaston’s rarity measure, rare plants had significantly higher positive associations with species across all three vegetation communities than common species. Positive associations between rare species with low abundances indicate that species may not be competing for the same resource [31], but rather occupy unique, narrow niches [51] that enable their persistence. Using Rabinowitz’s measure of rarity, plants classified as most rare, had no positive or negative associations with other species. Rabinowitz rare species with high habitat specificity do not interact or compete with other species within a community as their abundance correlated strongly with their environmental niche [52]. Thus, niche-based processes may be a key factor influencing the rarity of a species [14]. However, as rare species do not frequently appear within a community due to their low abundance, our ability to effectively determine whether habitat specificity or the associations between species, particularly a negative association influence rarity, is unlikely. Additionally, the growth form of a species did not influence the associations present between species. The influence of growth form on species rarity may vary in relation to the vegetation community examined. Some ecosystems have observed an association between species abundance and the growth form of surrounding species [30, 53, 54] while others have observed no association between local abundance and a species growth form [55]. Future work examining positive and negative associations of the species in this study at a greater geographic scale may provide further information on the local and landscape drivers of plant rarity.

Plants categorised under the other rarity types by Rabinowitz (Table 1) can have positive and negative associations, depending on the vegetation community in which they were present. Plants classified as having a narrow distribution, small population size, and low habitat specificity (NSU) had few negative associations with other species. Species with low habitat specificity may have a broadly available niche. However, their low abundance implies relatively poor competitive ability with other species for available resources. Species with a wide distribution, small population, and low habitat specificity (classified as WSU, the most common species in our dataset) had few positive associations in forest communities, where they were high in abundance. However, WSU species were less abundant in heath communities but had many positive associations with all other species. Heath communities tend to occur in shallow soils and areas of low productivity [39, 56]. The reduced availability of resources may inhibit WSU species from dominating heath sites and becoming common. Consequently, Rabinowitz’s rarity measures suggest that the availability and competition for available niches drive species commonness and different types of species rarity.

### Rare species clustering

Site-level spatial clustering of rare species did not differ across different vegetation communities for both measures of rarity. Rabinowitz’s measure of rarity indicated higher site-level clustering compared to Gaston’s measure of rarity (Gaston = 31% of sites; Rabinowitz = 59% of sites). As Rabinowitz’s measure of rarity considers habitat specificity, the frequent clustering of rare species indicates rare plants are specialists, having high niche specificity at the local scale. The clustering of many rare species at a site suggests niche differentiation between rare plants with high habitat specificity as they occupy different ecological spaces to minimise competition [57]. Rare species can often occupy the edges of environmental and functional niche space to avoid competition with dominant species [52]. However, interspecific competition between species for resources may be affecting plant rarity in sites where clustering was not observed for rare species. Alternatively, neutral processes may also have played a role in sites where rare species clustering was not apparent [58].

Neutral theory suggests that species interaction frequencies are proportional to their relative abundance [59]. Deviations from neutral theory correspond to the occurrence of positive or negative association between species, driven by niche-based processes [60]. If several specialist rare species are present for each substrate, then the associations between species within a community may be weak due to the neutral dynamics of equally well-adapted species [61]. Such neutral processes may mask our ability to detect possible associations between species [62] despite influencing a species’ rarity. Across time, the interplay between neutral and niche-based processes could influence the rarity of a species relative to changes in the local environment. Examining how neutral and niche-based processes influence positive and negative associations and clustering between species across time with an environmental disturbance can indicate the niche mechanism that drives the rarity of a species [58].

### Vegetation community characteristics

Comparison of woodland and forest sites suggests that rare plants are associated with vegetation community characteristics. In woodland communities, the number of rare species present was positively associated with species evenness (with Gaston’s rarity measure) but negatively associated with species diversity (with Rabinowitz’s measure). More rare species present at sites with high species evenness suggest there may be many different niches for species to occupy and low competition between species. Niche availability is an important factor influencing vegetation patterns at local scales [63]. This finding supports the idea that niche differentiation influences the occurrence of many rare species [14]. However, Rabinowitz rare species with high habitat specificity were negatively associated with species diversity in woodland sites. Species diversity is also associated with niche availability [64]. Rarity being associated negatively with species diversity suggests that only certain species can occupy the available niches present. Frequent fire is known to influence plant community composition in our study system [29], and could drive niche availability and competition between species. Consequently, changes in vegetation community composition and resource availability due to fire [29, 65] could influence the presence of rare species.

In our forest sites, the number of rare species present was associated positively with species diversity for both measures of rarity. Interestingly, woodland and forest plots in our study system are similar floristically [29], yet differ in the associations observed between rarity and vegetation community characteristics. Known differences between forest and woodland communities, such as canopy cover [40] and herbivory [66] in our study system, may also influence the presence of rare species. Previous work in our study system found burnt sites with high densities of large herbivores had a higher proportion of Bracken (*Pteridium esculentum*, a fern) present and limited recovery of non-bracken vegetation [66]. As such, sites with reduced herbivory after fire may allow for more rare plants in forest communities than woodland communities. Biotic associations between plant species also may shift from positive to negative over time with changes in the environment and influence the temporal rarity of a species across time and space.

Rare species in heath communities were not associated with species diversity, density, and evenness. Previous work found positive facilitative associations between species in harsh abiotic conditions of arid, alpine, and stress-prone Mediterranean environments [26, 67, 68]. Heath environments in southeast Australia are usually associated with poor soils and low soil productivity [39, 56]. Consequently, other biotic interactions such as facilitation may play a more substantial influence on plant rarity in heath communities.

### The importance of different rarity measures

Our study found that both definitions of rarity produced different results, which may have consequences in identifying factors that may influence rarity and the predict threatened status of a species [36]. Gaston’s rarity measure selects species present in the 25^th^ quantile of abundance across a study area. While this can provide insights on changes in species abundance across space and time, it does not consider that a species can be highly abundant in one area and sparsely distributed in other areas. Gaston’s measure can also select for vagrant species, rare exotic species or species that may be rare early in a succession following disturbance, but then become common with time since disturbance [34]. In contrast, Rabinowitz’s measure of rarity provides greater detail in understanding the abundance of a species in relation to its distribution and habitat specificity [33, 69]. Determining the habitat specificity and distribution of a species alongside information on abundance can indicate vulnerability to environmental change across different vegetation types [36]. Our results suggest that the combined use of different ecologically relevant indices for rarity can provide insights into the mechanisms driving plant rarity at a local and landscape scale and consequently inform conservation efforts at different spatial scales. Moving forward, expanding the definition of rarity to include temporal dynamics of biotic associations may enable us to determine the drivers of species rarity across time and effectively predict extinction risk in response to environmental change [70].

## Conclusion

Examining spatial associations between plants can help understand the processes that shape plant rarity. The differences in spatial associations across woodland, forest, and heath communities demonstrate the importance of habitat specificity and competition in shaping both the commonness and rarity of a species. Our work builds upon previous studies examining how associations between species can be an important indicator of the drivers of plant rarity [31, 71]. Increasing our current understanding of other biotic drivers underlying plant co-occurrence patterns is critical to better predicting responses to changes in species composition across different vegetation communities.

## Supporting information

S1 Appendix Plant list, for all known and identified species found across the 86 sites surveyed and their rarity classification. Table 1 contains the list of plant species found across the 86 sites with growth form and rarity classification across two different measures of rarity. Table 2 contains number of species classified under Gaston’s measure of rarity compared to their classification under Rabinowitz’s categorical classification of the seven forms of rarity.
(DOCX)

S2 Appendix Sensitivity analysis linear model outputs examining the number of positive and negative associations a species had in relation to their rarity. Sensitivity analysis model outputs of negative binomial generalised linear models examining the association between the rarity of a species and the number of positive and negative associations a rare species had with all other species. Model outputs were created from values obtained using the "quasiswap count" algorithm in the vegan package (Oksanen et al. 2019), and for two different probability thresholds (0.05 and 0.01).
(DOCX)

S3 Appendix Model outputs examining species rarity in relation to species diversity, evenness and density. Negative binomial generalised linear model outputs tested for the associations between vegetation community composition variables (species diversity, evenness and density) with the number of rare plants at a site, classified by Gaston and Rabinowitz, across the 86 sites surveyed.
(DOCX)
