# Supplementary material for: Spatial associations between plants and vegetation community characteristics provide insights into the processes influencing plant rarity
Source: PLoS One. 2021 Dec 20;16(12):e0260215. doi: 10.1371/journal.pone.0260215 (PMC8687526; doi:10.1371/journal.pone.0260215)
Supplement: S1 Appendix — Table 1 contains the list of plant species found across the 86 sites with growth form and rarity classification across two different measures of rarity. Table 2 contains number of species classified under Gaston’s measure of rarity compared to their classification under Rabinowitz’s categorical classification of the seven forms of rarity. (DOCX) [file pone.0260215.s001.docx]

**S1 Supplementary material.** **Plant list, for all known and identified species found across the 86 sites surveyed.**

Plant list for all the for all known and identified species found across the 86 sites surveyed (Table 1). Growth form data was collected from the TRY database (Kattge et al. 2011), and the growth forms were recategorised as herb/shrub to shrub, shrub/tree as tree for analysis.

All species were classified across two different types of rarity; Gaston's (1994) rarity by abundance, in which a species that falls below the 25^th^ quantile for average abundance across the sites was classified as rare and Rabinowitz's (1981) seven forms of rarity (See Table 1 in the main document for descriptions for each Rabinowitz rarity measure)*.*

**Table 1. List of plant species (with species authority) found across the 86 sites with growth form and rarity classification across two different measures of rarity.** With Gaston's measure of rarity, a rare species was classified as rare with 1 and 0 being common. With Rabinowitz's measure of rarity, the rarest species was classified as NSS.

| **Species** | **Family** | **Growth form** | **Rarity classification by abundance** | **Rabinowitz's seven forms of rarity classification** |
| --- | --- | --- | --- | --- |
| *Acacia implexa* (Benth.) | Fabaceae | shrub | 1 | NSU |
| *Acacia longifolia* Andrews | Fabaceae | shrub | 0 | NSU |
| *Acacia longissimi* (H.L.Wendl.) | Fabaceae | shrub | 1 | NSU |
| *Acacia suaveolens* (Sm.) Willd. | Fabaceae | shrub | 0 | NSU |
| *Acacia terminalis* (Salisb.) J.F.Macbr. | Fabaceae | shrub | 0 | NSU |
| *Acacia ulicifolia* (Salisb.) | Fabaceae | shrub | 0 | NSU |
| *Acianthus fornicates* R.Br. | Orchidaceae | herb | 1 | NSS |
| *Acianthus pusillus* D.L.Jones | Orchidaceae | herb | 1 | NSS |
| *Acianthus sp.* M.A.Clem. & D.L.Jones | Orchidaceae | herb | 1 | NSU |
| *Acmena smithii* (Poir.) Merr. & L.M.Perry | Myrtaceae | shrub | 1 | NSS |
| *Actinotus helianthin* Labill. | Apiaceae | shrub | 0 | NSU |
| *Actinotus minor* (Sm.) DC. | Apiaceae | herb | 0 | NSU |
| *Allocasuarina distyle* (Vent.) L.A.S.Johnson | Casuarinaceae | shrub | 0 | NSS |
| *Allocasuarina littoralis* (Salisb.) L.A.S.Johnson | Casuarinaceae | tree | 1 | NSS |
| *Aotus ericoides (Vent.) Don* | Fabaceae | shrub | 0 | NSS |
| *Austrostipa mollis* (R.Br.) S.W.L.Jacobs & J.Everett | Poaceae | graminoid | 0 | NSS |
| *Austrostipa pubescens* (R.Br.) S.W.L.Jacobs & J.Everett | Poaceae | graminoid | 0 | NSU |
| *Austrostipa sp.* (R.Br.) S.W.L.Jacobs & J.Everett | Poaceae | graminoid | 0 | NSU |
| *Baeckea brevifolia* (Rudge) DC. | Myrtaceae | shrub | 0 | NSS |
| *Baeckea imbricata* (Gaertn.) Druce | Myrtaceae | shrub | 0 | NSS |
| *Baeckea linifolia* Rudge | Myrtaceae | shrub | 0 | NSS |
| *Baloskion tetraphyllum* (Labill.) B.G.Briggs & L.A.S.Johnson | Restionaceae | herb | 1 | NSU |
| *Baloskion tetraphyllum subsp. Meiostachyum* (Labill.) B.G.Briggs & L.A.S.Johnson | Restionaceae | herb | 0 | NLS |
| *Banksia ericifolia* L.f. | Proteaceae | shrub | 0 | NSU |
| *Banksia integrifolia* L.f. | Proteaceae | tree | 1 | NSU |
| *Banksia integrifolia subsp. integrifolia* L.f. | Proteaceae | tree | 1 | NSS |
| *Banksia paludosa* R.Br. | Proteaceae | shrub | 0 | NSU |
| *Banksia serrata* L.f. | Proteaceae | shrub | 0 | NSU |
| *Banksia spinulosa* Sm. | Proteaceae | shrub | 1 | NSS |
| *Bauera rubioides* Andrews | Cunoniaceae | shrub | 0 | NSU |
| *Baumea acuta* (Labill.) J.Kern | Cyperaceae | graminoid | 0 | NSU |
| *Baumea juncea* (R.Br.) T.Koyama | Cyperaceae | graminoid | 0 | NSU |
| *Baumea sp.* (R.Br.) | Cyperaceae | graminoid | 1 | NSU |
| *Billardiera scandens* Sm. | Pittosporaceae | shrub | 1 | NSU |
| *Blandfordia nobilis* Sm. | Blandfordiaceae | herb | 1 | NSS |
| *Boronia barkeriana* F.Muell. | Rutaceae | shrub | 0 | NSS |
| *Boronia pinnata* Sm. | Rutaceae | shrub | 0 | NSU |
| *Bossiaea ensata* Sieber ex DC. | Fabaceae | shrub | 0 | NSU |
| *Bossiaea heterophylla* Vent. | Fabaceae | shrub | 0 | NSU |
| *Bossiaea scolopendria* (Andrews) Sm. | Fabaceae | shrub | 1 | NSS |
| *Breynia oblongifolia* Müll.Arg | Phyllanthaceae | shrub | 0 | NSS |
| *Burchardia umbellata* R.Br. | Colchicaceae | herb | 1 | NSU |
| *Caladenia carnea* R.B | Orchidaceae | herb | 0 | NSU |
| *Caladenia sp.* Hopper & Brown | Orchidaceae | herb | 1 | NSU |
| *Callicoma serratifolia* Andrews | Cunoniaceae | shrub | 1 | NSS |
| *Callistemon citrinus* (Curtis) Skeels | Myrtaceae | shrub | 0 | NSU |
| *Callistemon sp.* (R.Br.) M.D.Turner & R.A.White | Myrtaceae | shrub | 1 | NSS |
| *Calochlaena dubia* (R.Br.) M.D.Turner & R.A.White | Dicksoniaceae | fern | 0 | NSU |
| *Cassytha glabella* R.Br. | Lauraceae | herb | 1 | NSU |
| *Cassytha pubescens* R.Br. | Lauraceae | herb | 1 | NSS |
| *Cassytha sp.* R.Br. | Lauraceae | herb | 1 | NSS |
| *Casuarina glauca* Sieber ex Spreng. | Casuarinaceae | tree | 1 | NSU |
| *Caustis flexuosa* R.Br. | Cyperaceae | graminoid | 0 | NSU |
| *Caustis pentandra R.Br* | Cyperaceae | graminoid | 1 | NSU |
| *Caustis recurvata* Spreng. | Cyperaceae | graminoid | 0 | NSU |
| *Ceratopetalum gummiferum* Sm. | Cunoniaceae | shrub | 0 | NSS |
| *Chordifex fastigiatus* (R.Br.) | Restionaceae | herb | 0 | NSS |
| *Chorizema parviflorum* Benth. | Fabaceae | shrub | 1 | NSS |
| *Chrysanthemoides monilifera* (L.) Norl. | Asteraceae | shrub | 0 | NSS |
| *Cissus hypoglauca* A.Gray | Vitaceae | herb | 0 | NSS |
| *Clematis aristata* R.Br. ex Ker Gawl. | Ranunculaceae | herb | 0 | NSS |
| *Comesperma volubile* Labill. | Polygalaceae | herb | 0 | NSU |
| *Commelina cyanea* R.Br. | Commelinaceae | herb | 0 | NSS |
| *Commersonia hermanniifolia* J.Gay ex Kunth | Malvaceae | shrub | 1 | NSS |
| *Conospermum ericifolium* Sm. | Proteaceae | shrub | 1 | NSS |
| *Conyza sumatrensis* (Retz.) E.Walker | Asteraceae | herb | 1 | NSS |
| *Coronidium elatum* (A.Cunn. ex DC.) Paul G.Wilson | Asteraceae | shrub | 1 | NSU |
| *Correa reflexa var. reflexa* (Labill.) Vent. var. reflexa | Rutaceae | shrub | 1 | NSS |
| *Corybas sp.* D. L. Jones | Orchidaceae | herb | 1 | NSU |
| *Corymbia gummifera* (Gaertn.) K.D.Hill & L.A.S.Johnson | Myrtaceae | tree | 0 | NSU |
| *Corymbia sp.* | Myrtaceae | tree | 1 | NSU |
| *Cryptandra ericoides* Sm. | Rhamnaceae | shrub | 1 | NSS |
| *Cryptostylis erecta* F.Muell. ex Benth. | Orchidaceae | herb | 1 | NSU |
| *Cyathochaeta diandra* (R.Br.) Nees | Cyperaceae | graminoid | 0 | NSU |
| *Cyperus gracilis* R.Br. | Cyperaceae | graminoid | 0 | NSS |
| *Dampiera purpurea* R.Br. | Goodeniaceae | shrub | 1 | NSU |
| *Dampiera sp.* | Goodeniaceae | shrub | 1 | NSU |
| *Dampiera stricta* (Sm.) R.Br. | Goodeniaceae | shrub | 0 | NSU |
| *Darwinia camptostylis* B.G.Briggs | Myrtaceae | shrub | 0 | NSS |
| *Darwinia leptantha* B.G.Briggs | Myrtaceae | shrub | 0 | NSU |
| *Darwinia sp.* | Myrtaceae | shrub | 0 | NSU |
| *Pullenia gunnii* (Benth. ex Hook.f.) H.Ohashi & K.Ohashi | Fabaceae | herb | 0 | NSS |
| *Oxytes brachypoda* (A.Gray) H.Ohashi & K.Ohashi | Fabaceae | shrub | 1 | NSU |
| *Desmodium rhytidophyllum* (F.Muell.) Benth. | Fabaceae | shrub | 1 | NSU |
| *Desmodium varians* (Labill.) G.Don | Fabaceae | herb | 0 | NSU |
| *Dianella caerulea* Sims | Phormiaceae | herb | 0 | NSU |
| *Dichondra repens* J.R.Forst. & G.Forst. | Convolvulaceae | herb | 0 | NSU |
| *Dillwynia elegans* Endl. | Fabaceae | shrub | 0 | NSS |
| *Dillwynia floribunda* Sm. | Fabaceae | shrub | 0 | NSU |
| *Dillwynia floribunda var. floribunda* Sm. | Fabaceae | shrub | 0 | NSU |
| *Dillwynia glaberrima* Sm. | Fabaceae | shrub | 0 | NSU |
| *Dillwynia retorta* (J.C.Wendl.) Druce | Fabaceae | shrub | 0 | NSU |
| *Dillwynia sp.* | Fabaceae | shrub | 0 | NSU |
| *Dodonaea triquetra* J.C.Wendl. | Sapindaceae | shrub | 0 | NSS |
| *Drosera binata* Labill. | Droseraceae | herb | 0 | NSS |
| *Drosera peltata* Thunb. | Droseraceae | herb | 0 | NSU |
| *Drosera sp.* | Droseraceae | herb | 1 | NSU |
| *Drosera spatulata* Labill. | Droseraceae | herb | 1 | NSS |
| *Echinopogon ovatus* (G.Forst.) | Poaceae | graminoid | 1 | NSS |
| *Ehrharta erecta* Lam. | Poaceae | graminoid | 0 | NSS |
| *Elaeocarpus reticulatus* Sm. | Poaceae | tree | 0 | NSU |
| *Elaeodendron austral* (Vent.) Kuntze | Celastraceae | shrub | 1 | NSS |
| *Empodisma minus* (Hook.f.) | Restionaceae | herb | 0 | NSU |
| *Entolasia marginate* (R.Br.) Hughes | Poaceae | graminoid | 0 | WSU |
| *Entolasia stricta* (R.Br.) Hughes | Poaceae | graminoid | 0 | NSU |
| *Epacris longiflora* Cav. | Ericaceae | shrub | 1 | NSS |
| *Epacris microphylla* R.Br. | Ericaceae | shrub | 0 | NSU |
| *Epacris microphylla var. microphylla* R.Br. | Ericaceae | shrub | 0 | NSU |
| *Epacris obtusifolia* Sm. | Ericaceae | shrub | 0 | NSS |
| *Epacris pulchella* Cav. | Ericaceae | shrub | 0 | NSU |
| *Eragrostis brownii* (Kunth) Nees | Poaceae | graminoid | 1 | NSS |
| *Eragrostis sp.* | Poaceae | graminoid | 0 | NSS |
| *Eucalyptus botryoides* Sm. | Myrtaceae | tree | 1 | NSU |
| *Eucalyptus burgessiana* L.A.S.Johnson & Blaxell | Myrtaceae | tree | 0 | NSS |
| *Eucalyptus pilularis* Sm. | Myrtaceae | tree | 1 | NSU |
| *Eucalyptus racemosa* Cav. | Myrtaceae | tree | 1 | NSS |
| *Eurychorda complanata* (R.Br.) | Restionaceae | herb | 0 | NSS |
| *Eustrephus latifolius* R.Br. | Luzuriagaceae | shrub | 0 | NSS |
| *Ficinia nodosa* (Rottb.) Goetgh., Muasya & D.A.Simpson | Cyperaceae | graminoid | 0 | NSS |
| *Gahnia clarkei* Benl | Cyperaceae | graminoid | 0 | NSU |
| *Gahnia sieberiana* Kunth | Cyperaceae | graminoid | 0 | NSS |
| *Gahnia sp.* | Cyperaceae | graminoid | 1 | NSU |
| *Galium gaudichaudii* DC. | Rubiaceae | herb | 1 | NSS |
| *Galium leiocarpum* I.Thomps. | Rubiaceae | herb | 0 | NSU |
| *Gastrodia sesamoides* R.Br. | Orchidaceae | herb | 1 | NSS |
| *Geranium homeanum* Turcz. | Geraniaceae | herb | 0 | NSU |
| *Gleichenia dicarpa* R.Br. | Gleicheniaceae | fern | 0 | NSU |
| *Glycine clandestina* J.C.Wendl. | Fabaceae | herb | 0 | NSS |
| *Glycine tabacina* (Labill.) Benth. | Fabaceae | herb | 1 | NSS |
| *Gompholobium grandiflorum* Sm. | Fabaceae | shrub | 1 | NSS |
| *Gompholobium inconspicuum* Crisp | Fabaceae | shrub | 1 | NSS |
| *Gompholobium latifolium* Sm. | Fabaceae | shrub | 0 | NSS |
| *Gonocarpus micranthus* Thunb. | Haloragaceae | herb | 1 | NSS |
| *Gonocarpus micranthus subsp. micranthus* Thunb. | Haloragaceae | herb | 0 | NSU |
| *Gonocarpus sp.* | Haloragaceae | herb | 1 | NSS |
| *Gonocarpus teucrioides* DC. | Haloragaceae | herb | 0 | NSU |
| *Goodenia bellidifolia* Sm. | Goodeniaceae | herb | 0 | NSS |
| *Goodenia hederacea* Sm. | Goodeniaceae | herb | 1 | NSS |
| *Goodenia heterophylla subsp. eglandulosa* Carolin | Goodeniaceae | herb | 0 | NSS |
| *Goodenia stelligera* R.Br. | Goodeniaceae | herb | 1 | NSS |
| *Grevillea macleayana* (McGill.) Olde & Marriott | Proteaceae | shrub | 0 | NSS |
| *Grevillea sp.* | Proteaceae | shrub | 1 | NSS |
| *Gymnoschoenus sphaerocephalus* (R.Br.) Hook.f. | Cyperaceae | graminoid | 0 | NSS |
| *Haemodorum corymbosum* Vahl | Haemodoraceae | herb | 1 | NSU |
| *Haemodorum planifolium* R.Br. | Haemodoraceae | herb | 0 | NSU |
| *Hakea dactyloides* (Gaertn.) Cav. | Proteaceae | shrub | 0 | NSU |
| *Hakea sericea* Schrad. & J.C.Wendl. | Proteaceae | shrub | 0 | NSS |
| *Hakea teretifolia* (Salisb.) Britten | Proteaceae | shrub | 0 | NSU |
| *Hardenbergia violacea* (Schneev.) Stearn | Fabaceae | herb | 0 | NSU |
| *Hibbertia aspera* DC. | Dilleniaceae | shrub | 0 | NSS |
| *Hibbertia dentata* R.Br. ex DC. | Dilleniaceae | herb | 0 | NSS |
| *Hibbertia diffusa* DC. | Dilleniaceae | shrub | 0 | NSU |
| *Hibbertia empetrifolia* DC. | Dilleniaceae | shrub | 0 | NSU |
| *Hibbertia fasciculata* DC. | Dilleniaceae | shrub | 0 | NSS |
| *Hibbertia hermanniifolia* DC. | Dilleniaceae | shrub | 1 | NSS |
| *Hibbertia linearis* DC. | Dilleniaceae | shrub | 1 | NSU |
| *Hibbertia obtusifolia* DC. | Dilleniaceae | shrub | 1 | NSS |
| *Hibbertia riparia* (R.Br. ex DC.) Hoogland | Dilleniaceae | shrub | 0 | NSS |
| *Hibbertia scandens* (Willd.) Dryand. | Dilleniaceae | herb | 0 | NSU |
| *Hibbertia virgata* R.Br. ex DC. | Dilleniaceae | shrub | 1 | NSU |
| *Histiopteris incisa* (Thunb.) J.Sm. | Dennstaedtiaceae | fern | 1 | NSS |
| *Homalanthus populifolius* Graham | Euphorbiaceae | shrub | 1 | NSS |
| *Hovea linearis* (Sm.) R.Br. | Fabaceae | shrub | 1 | NSS |
| *Hybanthus monopetalus* (Schult.) Domin | Violaceae | herb | 1 | NSU |
| *Hybanthus vernoni* (F.Muell.) F.Muell. | Violaceae | herb | 1 | NSS |
| *Hybanthus vernonii subsp. scaber* E.M.Benn. | Violaceae | herb | 1 | NSU |
| *Hybanthus vernonii subsp. vernonii* (F.Muell.) F.Muell. | Violaceae | herb | 1 | NSU |
| *Hydrocotyle bonariensis* Lam. | Araliaceae | herb | 0 | NSS |
| *Hydrocotyle peduncularis* Lam. | Araliaceae | herb | 0 | NSS |
| *Hypochaeris radicata* L. | Asteraceae | herb | 1 | NSU |
| *Hypolaena fastigiate* R.Br. | Restionaceae | herb | 0 | NSU |
| *Hypolepis muelleri* N.A.Wakef. | Dennstaedtiaceae | fern | 1 | NSU |
| *Hypoxis hygrometrica var. hygrometrica* Labill. var. | Hypoxidaceae | herb | 1 | NSS |
| *Imperata cylindrica* (L.) P.Beauv. | Poaceae | graminoid | 0 | WSU |
| *Isopogon anemonifolius* (Salisb.) Knight | Proteaceae | shrub | 0 | NSU |
| *Isopogon anethifolius* (Salisb.) Knight | Proteaceae | shrub | 1 | NSU |
| *Isopogon sp.* | Proteaceae | shrub | 1 | NSU |
| *Juncus pallidus* R.Br. | Juncaceae | graminoid | 0 | NLS |
| *Juncus sp.* | Juncaceae | graminoid | 1 | NSU |
| *Juncus usitatus* L.A.S.Johnson | Juncaceae | graminoid | 0 | NSS |
| *Kennedia rubicunda* Vent. | Fabaceae | herb | 0 | NSU |
| *Kunzea capitata* (Sm.) Heynh. | Myrtaceae | shrub | 0 | NSS |
| *Lagenifera stipitata* (Labill.) Druce | Asteraceae | herb | 0 | NSS |
| *Lambertia formosa* Sm. | Proteaceae | shrub | 0 | NSU |
| *Leionema diosmium* (A.Juss.) Paul G.Wilson | Rutaceae | shrub | 0 | NLS |
| *Lepidosperma concavum* R.Br. | Cyperaceae | graminoid | 0 | NSU |
| *Lepidosperma filiforme* Labill. | Cyperaceae | graminoid | 0 | NSU |
| *Lepidosperma forsythia* A.A.Ham | Cyperaceae | graminoid | 0 | NSS |
| *Lepidosperma laterale* R.Br. | Cyperaceae | graminoid | 0 | NSS |
| *Lepidosperma longitudinale* Labill. | Cyperaceae | graminoid | 0 | NLS |
| *Lepidosperma urophorum* N.A.Wakef. | Cyperaceae | graminoid | 0 | NSS |
| *Leptocarpus tenax* (Labill.) R.Br. | Restionaceae | herb | 0 | NSU |
| *Leptomeria acida* R.Br. | Santalaceae | shrub | 1 | NSU |
| *Leptospermum continentale* Joy Thomps. | Myrtaceae | shrub | 0 | NSU |
| *Leptospermum epacridoideum* Cheel | Myrtaceae | shrub | 0 | NSS |
| *Leptospermum juniperinum* Sm. | Myrtaceae | shrub | 0 | NSU |
| *Leptospermum laevigatum* (Gaertn.) F.Muell. | Myrtaceae | shrub | 0 | NSU |
| *Leptospermum polygalifolium* Salisb. | Myrtaceae | shrub | 0 | NSU |
| *Leptospermum rotundifolium* (Maiden & Betche) F.A.Rodway | Myrtaceae | shrub | 0 | NSS |
| *Leptospermum squarrosum* Gaertn. | Myrtaceae | shrub | 1 | NSS |
| *Leptospermum trinervium* (Sm.) Joy Thomps. | Myrtaceae | shrub | 0 | NSU |
| *Lepyrodia scariosa* R.Br. | Restionaceae | herb | 0 | NSU |
| *Leucopogon ericoides* (Sm.) R.Br. | Ericaceae | shrub | 1 | NSU |
| *Leucopogon esquamatus* R.Br. | Ericaceae | shrub | 0 | NSS |
| *Leucopogon lanceolatus* (Sm.) R.Br. | Ericaceae | shrub | 0 | NSU |
| *Leucopogon parviflorus* (Andrews) Lindl. | Ericaceae | shrub | 1 | NSS |
| *Lindsaea linearis* Sw. | Lindsaeaceae | fern | 0 | NSU |
| *Livistona australis* (R.Br.) Mart. | Arecaceae | tree | 1 | NSS |
| *Lobelia anceps* L.f. | Campanulaceae | herb | 1 | NSS |
| *Lobelia dentata* Cav. | Campanulaceae | herb | 1 | NSU |
| *Lobelia purpurascens* R.Br. | Campanulaceae | herb | 0 | NSS |
| *Lomandra cylindrica* A.T.Lee | Lomandraceae | herb | 0 | NSU |
| *Lomandra filiformis* (Thunb.) Britten | Lomandraceae | herb | 0 | NSS |
| *Lomandra filiformis* (Thunb.) Britten subsp. *filiformis* | Lomandraceae | herb | 0 | NSU |
| *Lomandra glauca* (R.Br.) Ewart | Lomandraceae | herb | 0 | NSU |
| *Lomandra gracilis* (R.Br.) A.T.Lee | Lomandraceae | herb | 0 | NSU |
| *Lomandra longifolia* Labill. | Lomandraceae | herb | 0 | WSU |
| *Lomandra multiflora* (R.Br.) Britten subsp. *multiflora* | Lomandraceae | herb | 0 | NSU |
| *Lomandra obliqua* (Thunb.) J.F.Macbr. | Lomandraceae | herb | 0 | NSU |
| *Lomatia ilicifolia* R.Br. | Proteaceae | shrub | 1 | NSS |
| *Lycopodium deuterodensum* Herter | Lycopodiaceae | fern | 0 | NLS |
| *Lycopodium laterale* (R.Br.) B.Øllg. | Lycopodiaceae | fern | 0 | NSS |
| *Lysimachia arvensis* (L.) U.Manns & Anderb. | Primulaceae | herb | 1 | NSS |
| *Marsdenia rostrata* R.Br. | Apocynaceae | herb | 0 | NSU |
| *Marsdenia sp.* | Apocynaceae | herb | 1 | NSU |
| *Marsdenia suaveolens* R.Br. | Apocynaceae | herb | 0 | NSS |
| *Melaleuca capitata* Cheel | Myrtaceae | shrub | 0 | NLS |
| *Melaleuca linariifolia* Sm. | Myrtaceae | tree | 1 | NSS |
| *Melaleuca sieberi* Schauer | Myrtaceae | shrub | 0 | NSS |
| *Melaleuca sp.* | Myrtaceae | shrub | 1 | NSS |
| *Melaleuca squarrosa* Donn ex Sm. | Myrtaceae | shrub | 0 | NSS |
| *Melaleuca thymifolia* Sm. | Myrtaceae | shrub | 0 | NSS |
| *Microlaena stipoides* (Labill.) R.Br. var. *stipoides* | Myrtaceae | graminoid | 0 | NSU |
| *Mirbelia rubiifolia* (Andr.) G.Don | Fabaceae | shrub | 0 | NSS |
| *Mitrasacme polymorpha* R.Br. | Loganiaceae | herb | 1 | NSU |
| *Monotoca elliptica* (Sm.) R.Br. | Ericaceae | shrub | 0 | NSU |
| *Monotoca scoparia* (Sm.) R.Br. | Ericaceae | shrub | 1 | NSU |
| *Myoporum acuminatum* R.Br. | Scrophulariaceae | shrub | 1 | NSU |
| *Notelaea longifolia* Vent. | Oleaceae | shrub | 0 | NSS |
| *Notelaea longifolia* Vent. f. *longifolia* | Oleaceae | shrub | 1 | NSS |
| *Notelaea ovata* R.Br. | Oleaceae | shrub | 0 | NSS |
| *Notelaea sp.* | Oleaceae | shrub | 1 | NSU |
| *Notelaea venosa* F.Muell. | Oleaceae | shrub | 0 | NSS |
| *Opercularia aspera* Gaertn. | Rubiaceae | shrub | 0 | NSU |
| *Opercularia diphylla* Gaertn. | Rubiaceae | shrub | 0 | NSU |
| *Opercularia hispida* Spreng. | Rubiaceae | shrub | 1 | NSU |
| *Opercularia varia* Hook.f. | Rubiaceae | shrub | 0 | NSS |
| *Oplismenus aemulus* (R.Br.) Roem. & Schult. | Poaceae | graminoid | 0 | NSS |
| *Oplismenus imbecillis* (R.Br.) Roem. & Schult. | Poaceae | graminoid | 1 | NSS |
| *Oxalis articulate* Savigny | Oxalidaceae | herb | 0 | NSS |
| *Oxalis corniculate* L. | Oxalidaceae | herb | 0 | NSS |
| *Oxalis perennans* Haw. | Oxalidaceae | herb | 1 | NSS |
| *Oxalis sp.* | Oxalidaceae | herb | 0 | NSS |
| *Pandorea pandorana* (Andrews) Steenis | Bignoniaceae | herb | 0 | NSS |
| *Parsonsia straminea* (R.Br.) F.Muell. | Apocynaceae | herb | 1 | NSU |
| *Patersonia fragilis* (Labill.) Asch. & Graebn. | Iridaceae | herb | 0 | NSS |
| *Patersonia glabrata* R.Br. | Iridaceae | herb | 0 | NSU |
| *Patersonia sericea* R.Br. | Iridaceae | herb | 0 | NSU |
| *Pelargonium inodorum* Willd. | Geraniaceae | herb | 1 | NSS |
| *Persoonia levis* (Cav.) Domin | Proteaceae | shrub | 1 | NSU |
| *Persoonia linearis* Andrews | Proteaceae | shrub | 1 | NSU |
| *Persoonia mollis* subsp. *caleyi* (R.Br.) S.L.Krauss & L.A.S.Johnson | Proteaceae | shrub | 1 | NSU |
| *Petrophile pedunculata* R.Br. | Proteaceae | shrub | 1 | NSS |
| *Petrophile pulchella* Schrad. & J.C.Wendl.) R.Br. | Proteaceae | shrub | 1 | NSU |
| *Petrophile sessilis* ex Schult. & Schult.f. | Proteaceae | shrub | 0 | NSU |
| *Philotheca buxifolia* (Sm.) Paul G.Wilson | Rutaceae | shrub | 0 | NSU |
| *Phyllota phylicoides* (Sieber ex DC.) Benth. | Fabaceae | shrub | 1 | NSS |
| *Pimelea ligustrina* Labill. | Thymelaeaceae | shrub | 0 | NSU |
| *Pimelea linifolia* Sm. | Thymelaeaceae | shrub | 1 | NSU |
| *Pittosporum revolutum* Dryand. ex W.T.Aiton | Pittosporaceae | shrub | 1 | NSS |
| *Pittosporum undulatum* Vent. | Pittosporaceae | shrub | 0 | NSS |
| *Platylobium formosum* Sm. | Fabaceae | shrub | 0 | NSU |
| *Platysace lanceolata* (Labill.) Druce | Apiaceae | shrub | 1 | NSU |
| *Poa labillardierei* Steud. | Poaceae | graminoid | 0 | NSU |
| *Poa sp.* | Poaceae | graminoid | 1 | NSS |
| *Polyscias sambucifolia* (Sieber ex DC.) Harms | Araliaceae | shrub | 1 | NSS |
| *Pomax umbellata* (Gaertn.) Sol. ex A.Rich. | Rubiaceae | shrub | 0 | NSS |
| *Poranthera ericifolia* Rudge | Phyllanthaceae | shrub | 1 | NSU |
| *Poranthera microphylla* Brongn. | Phyllanthaceae | herb | 0 | NSU |
| *Prasophyllum brevilabre* (Lindl.) Hook.f. | Orchidaceae | herb | 1 | NSS |
| *Psychotria loniceroides* Sieber ex DC. | Rubiaceae | shrub | 1 | NSS |
| *Pteridium esculentum* (G.Forst.) Cockayne | Dennstaedtiaceae | fern | 0 | WSU |
| *Pterostylis sp.* | Orchidaceae | herb | 1 | NSU |
| *Ptilothrix deusta* (R.Br.) K.L.Wilson | Cyperaceae | graminoid | 0 | NSS |
| *Pultenaea daphnoides* J.C.Wendl. | Fabaceae | shrub | 0 | NSU |
| *Pultenaea rosmarinifolia* Lindl. | Fabaceae | shrub | 1 | NSU |
| *Pultenaea villifera* DC. | Fabaceae | shrub | 1 | NSS |
| *Pultenaea villosa Willd.* | Fabaceae | shrub | 0 | NSS |
| *Sarcopetalum harveyanum* F.Muell. | Menispermaceae | shrub | 1 | NSS |
| *Scaevola ramosissima* (Sm.) K.Krause | Goodeniaceae | herb | 0 | NSU |
| *Schelhammera undulata* R.Br. | Uvulariaceae | herb | 0 | NSS |
| *Schizaea bifida* Willd. | Schizaeaceae | fern | 1 | NSU |
| *Schizaea dichotoma* (L.) Sm. | Schizaeaceae | fern | 1 | NSS |
| *Schoenus ericetorum* R.Br. | Cyperaceae | graminoid | 0 | NSS |
| *Schoenus pachylepis*S.T.Blake | Cyperaceae | graminoid | 0 | NLS |
| *Selaginella uliginosa* (Labill.) Spring | Selaginellaceae | fern | 0 | NSU |
| *Smilax glyciphylla* Sm. | Smilacaceae | shrub | 0 | NSU |
| *Solanum nigrum* L. | Solanaceae | herb | 1 | NSS |
| *Solanum prinophyllum* Dunal | Solanaceae | herb | 1 | NSU |
| *Solanum stelligerum* Sm. | Solanaceae | herb | 1 | NSS |
| *Sowerbaea juncea* Andrews | Anthericaceae | herb | 0 | NSS |
| *Sphaerolobium vimineum* Sm. | Fabaceae | shrub | 1 | NSS |
| *Sprengelia incarnata* Sm. | Ericaceae | shrub | 0 | NSS |
| *Stackhousia nuda* Lindl. | Stackhousiaceae | herb | 0 | NSS |
| *Stephania japonica var. discolour* (Blume) Forman | Menispermaceae | herb | 0 | NSS |
| *Stylidium laricifolium* Rich. | Stylidiaceae | shrub | 0 | NSU |
| *Stylidium lineare* Sw. ex Willd. | Stylidiaceae | herb | 1 | NSS |
| *Styphelia trifloral* Andrews | Ericaceae | shrub | 1 | NSU |
| *Styphelia tubiflora* Sm. | Myrtaceae | shrub | 1 | NSS |
| *Syncarpia glomulifera* (Sm.) Nied. | Meliaceae | tree | 0 | NSU |
| *Synoum glandulosum* (Sm.) A.Juss. | Meliaceae | tree | 0 | NSU |
| *Syzygium paniculatum* Gaertn. | Myrtaceae | shrub | 0 | NSS |
| *Telopea speciosissima* (Sm.) R.Br. | Proteaceae | shrub | 0 | NSU |
| *Tetratheca thymifolia* Sm. | Elaeocarpaceae | shrub | 1 | NSS |
| *Thelionema umbellatum* (R.Br.) R.J.F.Hend. | Phormiaceae | herb | 1 | NSS |
| *Themeda triandra* Forssk. | Poaceae | graminoid | 1 | NSU |
| *Thysanotus juncifolius* (Salisb.) J.H.Willis & Court | Anthericaceae | herb | 0 | NSS |
| *Thysanotus tuberosus* R.Br. | Anthericaceae | herb | 0 | NSU |
| *Tristaniopsis collina* Peter G.Wilson & J.T.Waterh. | Myrtaceae | shrub | 1 | NSS |
| *Urtica incisa* Poir. | Urticaceae | herb | 0 | NSS |
| *Utricularia dichotoma* Labill. | Lentibulariaceae | herb | 1 | NSS |
| *Utricularia lateriflora* R.Br. | Lentibulariaceae | herb | 1 | NSS |
| *Veronica calycina* R.Br. | Plantaginaceae | herb | 1 | NSU |
| *Viminaria juncea* (Schrad.) Hoffmanns. | Fabaceae | shrub | 0 | NSU |
| *Viola hederacea* Labill. | Violaceae | herb | 0 | NSU |
| *Woollsia pungens* (Cav.) F.Muell. | Ericaceae | shrub | 0 | NSS |
| *Xanthorrhoea arborea* R.Br. | Xanthorrhoeaceae | shrub | 1 | NSU |
| *Xanthorrhoea australis* R.Br. | Xanthorrhoeaceae | shrub | 0 | NSS |
| *Xanthorrhoea minor subsp. lutea* | Xanthorrhoeaceae | shrub | 1 | NSS |
| *Xanthorrhoea resinifera* D.J.Bedford | Xanthorrhoeaceae | shrub | 0 | NSU |
| *Xanthorrhoea resinosa* Pers. | Xanthorrhoeaceae | shrub | 0 | NSS |
| *Xanthorrhoea sp.* | Xanthorrhoeaceae | shrub | 0 | NSU |
| *Xanthosia Pilosa* Rudge | Xanthorrhoeaceae | shrub | 0 | NSU |
| *Xanthosia tridentate* DC. | Apiaceae | shrub | 1 | NSS |
| *Xyris gracilis* R.Br. | Xyridaceae | herb | 1 | NSS |
| *Xyris operculate* Labill. | Xyridaceae | herb | 0 | NSS |
| *Xyris sp.* | Xyridaceae | herb | 0 | NSS |
| *Zieria Pilosa* Rudge | Rutaceae | shrub | 0 | NSS |
| *Zieria smithii* | Poaceae | shrub | 0 | NSS |

**Correlaton between both rarity indicies**

We found a significant correlation between species classified as rare by Gaston to a species classified by Rabinowitz’s measure of (chi-squared test, P = 0.0005).

**Table 2. The number of species classified under Gaston’s measure of rarity compared to their classification under Rabinowitz’s categorical classification of the seven forms of rarity.**

|  |  | Rabinowitz's measure of rarity categories | | | |
| --- | --- | --- | --- | --- | --- |
|  |  | NLS | NSS | NSU | WSU |
| Gaston's measure of rarity | Common | 8 | 104 | 108 | 4 |
|  | Rare | 0 | 158 | 73 | 0 |
