# Supplementary material for: Spatial associations between plants and vegetation community characteristics provide insights into the processes influencing plant rarity
Source: PLoS One. 2021 Dec 20;16(12):e0260215. doi: 10.1371/journal.pone.0260215 (PMC8687526; doi:10.1371/journal.pone.0260215)
Supplement: S2 Appendix — Sensitivity analysis model outputs of negative binomial generalised linear models examining the association between the rarity of a species and the number of positive and negative associations a rare species had with all other species. Model outputs were created from values obtained using the "quasiswap count" algorithm in the vegan package (Oksanen et al. 2019), and for two different probability thresholds (0.05 and 0.01). (DOCX) [file pone.0260215.s002.docx]

**S2 Supplementary Material. Sensitivity analysis linear model outputs examining the number of positive and negative associations a species had in relation to their rarity.**

Sensitivity analysis model outputs of negative binomial generalised linear models examining the association between the rarity of a species and the number of positive and negative associations a rare species had with all other species. Model outputs were created from values obtained using the "quasiswap count" algorithm in the vegan package (Oksanen et al. 2019), and for two different probability thresholds (0.05 and 0.01).

**Table 1. Model outputs for the associations a Gaston rare species had with other species using ‘quasiswap count’ and a 0.05 probability threshold.**Negative binomial generalised linear model outputs tested for the association between rarity of a species, classified with Gaston's measure of rarity, and the number of positive and negative associations the species had with all other species. Raw estimates, standardised regression coefficients and estimated 95% confidence intervals are shown. The reference level (intercept) are species considered common by Gaston's measure of rarity. In the model examining the number of positive associations in forest communities with rarity, the reference level (intercept) is the growth form, fern.

| **Vegetation community** | **Woodland** | | | | | | | | | | | | **Heath** | | | | | | | | **Forest** | | | | | | |
| --- | --- | --- | --- | --- | --- | --- | --- | --- | --- | --- | --- | --- | --- | --- | --- | --- | --- | --- | --- | --- | --- | --- | --- | --- | --- | --- | --- |
| **Type of association** | **No. of positive associations** | | | | | | **No. of negative associations** | | | | | | **No. of positive associations** | | | | **No. of negative associations** | | | | **No. of positive associations** | | | | **No. of negative associations** | | |
| *Predictors* | *Log-Mean* | | *CI* | | *p* | | *Log-Mean* | | *CI* | | *p* | | *Log-Mean* | | *CI* | *p* | *Log-Mean* | *CI* | *p* | *Log-Mean* | | *CI* | *p* | *Log-Mean* | | *CI* | *p* |
| (Intercept) | 2.39 | | 2.31 – 2.46 | | **<0.001** | | 1.92 | | 0.56 – 3.28 | | **0.006** | | 2.33 | | 2.25 – 2.41 | **<0.001** | -0.34 | -0.78 – 0.10 | 0.127 | 2.29 | | 2.19 – 2.40 | **<0.001** | 1.98 | | 1.72 – 2.24 | **<0.001** |
| Rare Gaston | -0.03 | | -0.15 – 0.09 | | 0.626 | | -22.22 | | -17609.18 – 17564.73 | | 0.998 | | -0.08 | | -0.23 – 0.07 | 0.294 | -20.96 | -6673.09 – 6631.17 | 0.995 | -0.33 | | -0.52 – -0.15 | **0.001** | -4.83 | | -6.04 – -3.62 | **<0.001** |
| Growth Form [graminoid] |  | |  | |  | | 0.10 | | -1.49 – 1.68 | | 0.904 | |  | |  |  |  |  |  |  | |  |  |  | |  |  |
| Growth Form [herb] |  | |  | |  | | -0.62 | | -2.12 – 0.89 | | 0.420 | |  | |  |  |  |  |  |  | |  |  |  | |  |  |
| Growth Form [shrub] |  | |  | |  | | -0.68 | | -2.11 – 0.75 | | 0.349 | |  | |  |  |  |  |  |  | |  |  |  | |  |  |
| Growth Form [tree] |  | |  | |  | | -2.33 | | -5.03 – 0.37 | | 0.091 | |  | |  |  |  |  |  |  | |  |  |  | |  |  |
|  |  | |  | |  | |  | |  | |  | |  | |  |  |  |  |  |  | |  |  |  | |  |  |
| Growth Form [graminoid] * Rare Gaston | |  | |  | |  | | -0.10 | | -21539.62 – 21539.43 | | 1.000 | |  |  |  |  |  |  |  |  |  |  |  |  |  |  |
| Growth Form [herb] * Rare Gaston | |  | |  | |  | | 19.42 | | -17567.53 – 17606.37 | | 0.998 | |  |  |  |  |  |  |  |  |  |  |  |  |  |  |
| Growth Form [shrub] * Rare Gaston | |  | |  | |  | | 0.68 | | -18127.53 – 18128.90 | | 1.000 | |  |  |  |  |  |  |  |  |  |  |  |  |  |  |
| Growth Form [tree] * Rare Gaston | |  | |  | |  | | 2.33 | | -21018.10 – 21022.75 | | 1.000 | |  |  |  |  |  |  |  |  |  |  |  |  |  |  |
| Observations | 198 | | | | | | 198 | | | | | | 181 | | | | 181 | | | | 147 | | | | 147 | | |
| R^2^ Nagelkerke | 0.002 | | | | | | 0.661 | | | | | | 0.010 | | | | 0.356 | | | | 0.114 | | | | 0.724 | | |

**Table 2. Model outputs for the associations a Gaston rare species had with other species using ‘quasiswap count’ and a 0.01 probability threshold.**Negative binomial generalised linear model outputs tested for the association between rarity of a species, classified with Gaston's measure of rarity, and the number of positive and negative associations the species had with all other species. A positive association or negative association was considered significant where the associated p-values in any of the two tests were lower than the *0.01* probability threshold. Raw estimates, standardised regression coefficients and estimated 95% confidence intervals are shown. The reference level (intercept) are species considered common by Gaston's measure of rarity. In the model examining the number of positive associations in forest communities with rarity, the reference level is (intercept) is the growth form, fern. *Note there were not enough negative associations between species detected in heath sites with the 0.01 probability threshold to text if rare species were associated with negative associations.*

| **Vegetation community** | **Woodland** | | | | | | **Heath** | | | **Forest** | | | | | | |
| --- | --- | --- | --- | --- | --- | --- | --- | --- | --- | --- | --- | --- | --- | --- | --- | --- |
| **Type of association** | **No. of positive associations** | | | **No. of negative associations** | | | **No. of positive associations** | | | **No. of negative associations** | | | | **No. of positive associations** | | |
| *Predictors* | *Log-Mean* | *CI* | *p* | *Log-Mean* | *CI* | *p* | *Log-Mean* | *CI* | *p* | *Log-Mean* | *CI* | *p* | *Log-Mean* | | *CI* | *p* |
| (Intercept) | 1.21 | 0.82 – 1.61 | **<0.001** | -0.32 | -0.82 – 0.18 | 0.212 | 1.03 | 0.32 – 1.74 | **0.004** | 1.56 | 1.44 – 1.68 | **<0.001** | 0.64 | | 0.26 – 1.02 | **0.001** |
| Growth Form [graminoid] | 0.03 | -0.43 – 0.49 | 0.900 |  |  |  | 0.51 | -0.27 – 1.28 | 0.200 |  |  |  |  | |  |  |
| Growth Form [herb] | 0.24 | -0.18 – 0.67 | 0.254 |  |  |  | 0.76 | 0.01 – 1.51 | **0.047** |  |  |  |  | |  |  |
| Growth Form [shrub] | 0.23 | -0.17 – 0.64 | 0.257 |  |  |  | 0.44 | -0.30 – 1.17 | 0.242 |  |  |  |  | |  |  |
| Growth Form [tree] | -0.32 | -0.94 – 0.31 | 0.317 |  |  |  | 0.07 | -1.64 – 1.78 | 0.937 |  |  |  |  | |  |  |
| Rare Gaston | -0.14 | -0.31 – 0.02 | 0.093 | -20.98 | -5781.90 – 5739.93 | 0.994 | 0.27 | -0.84 – 1.38 | 0.634 | -0.34 | -0.56 – -0.12 | **0.003** | -20.94 | | -4328.85 – 4286.96 | 0.992 |
| Growth Form [graminoid] * Rare Gaston |  |  |  |  |  |  | 0.50 | -0.95 – 1.94 | 0.502 |  |  |  |  | |  |  |
| Growth Form [herb] * Rare Gaston |  |  |  |  |  |  | -0.78 | -1.98 – 0.41 | 0.198 |  |  |  |  | |  |  |
| Growth Form [shrub] * Rare Gaston |  |  |  |  |  |  | -0.57 | -1.74 – 0.60 | 0.338 |  |  |  |  | |  |  |
| Growth Form [tree] * Rare Gaston |  |  |  |  |  |  | 0.93 | -1.17 – 3.04 | 0.384 |  |  |  |  | |  |  |
| Observations | 186 | | | 186 | | | 162 | | | 144 | | | | 144 | | |
| R^2^ | 0.091 | | | 0.437 | | | 0.182 | | | 0.092 | | | | 0.524 | | |

**Table 3. Model outputs for the associations a Rabinowitz rare species had with other species using ‘quasiswap count’ and a 0.05 probability threshold.**Negative binomial generalised linear model outputs tested for the association between rarity of a species, classified with Rabinowitz's measure of rarity, and the number of positive and negative associations the species had with all other species. A positive association or negative association was considered significant where the associated p-values in any of the two tests were lower than the *0.05* probability threshold. Raw estimates, standardised regression coefficients and estimated 95% confidence intervals are shown. The reference level (intercept) are species classified as NLS by Rabinowitz's measure of rarity. In the model examining the number of positive associations in forest communities with rarity, the reference level is (intercept) is the growth form, fern.

| **Vegetation community** | **Woodland** | | | | | | **Heath** | | | | | | **Forest** | | | | | |
| --- | --- | --- | --- | --- | --- | --- | --- | --- | --- | --- | --- | --- | --- | --- | --- | --- | --- | --- |
| **Type of association** | **No. of positive associations** | | | **No. of negative associations** | | | **No. of positive associations** | | | **No. of negative associations** | | | **No. of positive associations** | | | **Type of association** | | |
| *Predictors* | *Log-Mean* | *CI* | *p* | *Log-Mean* | *CI* | *p* | *Log-Mean* | *CI* | *p* | *Log-Mean* | *CI* | *p* | *Log-Mean* | *CI* | *p* | *Log-Mean* | *CI* | *p* |
| (Intercept) | 3.00 | 2.29 – 3.74 | **<0.001** | 2.89 | 0.08 – NA | 0.249 | 2.01 | 1.63 – 2.40 | **<0.001** | 0.98 | -0.70 – 4.23 | 0.379 | 1.77 | 1.26 – 2.28 | **<0.001** | 2.94 | 0.51 – 11.25 | 0.134 |
| Rare Rabinowitz  [NSS] | -0.71 | -1.47 – 0.00 | 0.055 | -2.73 | NA – 0.26 | 0.282 | 0.20 | -0.20 – 0.61 | 0.330 | -1.85 | -5.16 – 0.07 | 0.120 |  |  |  | -1.26 | -9.58 – 1.26 | 0.524 |
| Rare Rabinowitz  [NSU] | -0.58 | -1.33 – 0.13 | 0.117 | -2.00 | NA – 0.86 | 0.426 | 0.33 | -0.06 – 0.73 | 0.096 | -1.83 | -5.11 – -0.03 | 0.113 | 0.29 | 0.10 – 0.48 | **0.002** | -1.69 | -10.00 – 0.80 | 0.393 |
| Rare Rabinowitz  [WSU] | -0.60 | -1.45 – 0.22 | 0.157 | -0.67 | NA – 4.33 | 0.812 | 0.76 | 0.21 – 1.31 | **0.007** | -1.27 | -5.17 – 3.50 | 0.488 | 0.78 | 0.26 – 1.33 | **0.004** | -0.08 | -8.50 – 3.67 | 0.970 |
| Growth Form [graminoid] |  |  |  |  |  |  |  |  |  |  |  |  | 0.02 | -0.52 – 0.56 | 0.929 |  |  |  |
| Growth Form [herb] |  |  |  |  |  |  |  |  |  |  |  |  | 0.35 | -0.17 – 0.86 | 0.181 |  |  |  |
| Growth Form [shrub] |  |  |  |  |  |  |  |  |  |  |  |  | 0.19 | -0.33 – 0.70 | 0.477 |  |  |  |
| Growth Form [tree] |  |  |  |  |  |  |  |  |  |  |  |  | -0.01 | -0.61 – 0.58 | 0.965 |  |  |  |
| Observations | 198 | | | 198 | | | 181 | | | 181 | | | 146 | | | 147 | | |
| R^2^ | 0.049 | | | 0.058 | | | 0.092 | | | 0.061 | | | 0.184 | | | 0.061 | | |

**Table 4. Model outputs for the associations a Rabinowitz rare species had with other species using ‘quasiswap count’ and a 0.01 probability threshold.**
Negative binomial generalised linear model outputs tested for the association between rarity of a species, classified with Rabinowitz's measure of rarity, and the number of positive and negative associations the species had with all other species. A positive association or negative association was considered significant where the associated p-values in any of the two tests were lower than the *0.01* probability threshold. Raw estimates, standardised regression coefficients and estimated 95% confidence intervals are shown. The reference level (intercept) are species classified as NLS by Rabinowitz's measure of rarity. In the model examining the number of positive associations in forest communities with rarity, the reference level is (intercept) is the growth form, fern.

| **Vegetation community** | **Woodland** | | | | | | **Heath** | | | | | | **Forest** | | | | | |
| --- | --- | --- | --- | --- | --- | --- | --- | --- | --- | --- | --- | --- | --- | --- | --- | --- | --- | --- |
| **Type of association** | **No. of positive associations** | | | **No. of negative associations** | | | **No. of positive associations** | | | **No. of negative associations** | | | **No. of positive associations** | | | **Type of association** | | |
| *Predictors* | *Log-Mean* | *CI* | *p* | *Log-Mean* | *CI* | *p* | *Log-Mean* | *CI* | *p* | *Log-Mean* | *CI* | *p* | *Log-Mean* | *CI* | *p* | *Log-Mean* | *CI* | *p* |
| (Intercept) | 2.20 | 1.47 – 2.93 | **<0.001** | 1.39 | -5.22 – 7.99 | 0.681 | 1.72 | 1.07 – 2.37 | **<0.001** | 0.39 | -0.32 – 1.09 | 0.283 | 1.83 | 0.67 – 3.00 | **0.002** | 1.39 | -3.16 – 5.93 | 0.550 |
| Rare Rabinowitz [NSS] | -0.82 | -1.57 – -0.08 | **0.031** | -2.95 | -9.66 – 3.76 | 0.389 | -0.45 | -1.14 – 0.23 | 0.196 |  |  |  | -0.37 | -1.43 – 0.69 | 0.496 | -1.00 | -5.59 – 3.59 | 0.669 |
| Rare Rabinowitz [NSU] | -0.86 | -1.59 – -0.12 | **0.022** | -2.22 | -8.86 – 4.41 | 0.512 | -0.16 | -0.82 – 0.51 | 0.646 | -0.70 | -1.61 – 0.22 | 0.134 | -0.10 | -1.16 – 0.96 | 0.853 | -1.70 | -6.27 – 2.88 | 0.467 |
| Rare Rabinowitz [WSU] | -0.59 | -1.49 – 0.32 | 0.204 | -0.41 | -8.04 – 7.23 | 0.917 | 0.03 | -0.94 – 1.00 | 0.957 |  |  |  | 0.09 | -1.10 – 1.28 | 0.881 | 0.06 | -5.02 – 5.14 | 0.981 |
| Growth Form [graminoid] |  |  |  |  |  |  |  |  |  |  |  |  | -0.33 | -0.90 – 0.23 | 0.248 |  |  |  |
| Growth Form [herb] |  |  |  |  |  |  |  |  |  |  |  |  | -0.04 | -0.56 – 0.48 | 0.874 |  |  |  |
| Growth Form [shrub] |  |  |  |  |  |  |  |  |  |  |  |  | -0.22 | -0.75 – 0.31 | 0.413 |  |  |  |
| Growth Form [tree] |  |  |  |  |  |  |  |  |  |  |  |  | -0.77 | -1.43 – -0.11 | **0.022** |  |  |  |
| Observations | 183 | | | 183 | | | 163 | | | 139 | | | 144 | | | 144 | | |
| R^2^ | 0.043 | | | 0.066 | | | 0.056 | | | 0.036 | | | 0.183 | | | 0.074 | | |
