# Supplementary material for: Spatial associations between plants and vegetation community characteristics provide insights into the processes influencing plant rarity
Source: PLoS One. 2021 Dec 20;16(12):e0260215. doi: 10.1371/journal.pone.0260215 (PMC8687526; doi:10.1371/journal.pone.0260215)
Supplement: S3 Appendix — Negative binomial generalised linear model outputs tested for the associations between vegetation community composition variables (species diversity, evenness and density) with the number of rare plants at a site, classified by Gaston and Rabinowitz, across the 86 sites surveyed. (DOCX) [file pone.0260215.s003.docx]

**S3 Supplementary material. Model outputs examining species rarity in relation to species diversity, evenness and density.**Negative binomial generalised linear model outputs tested for the associations between vegetation community composition variables (species diversity, evenness and density) with the number of rare plants at a site, classified by Gaston and Rabinowitz, across the 86 sites surveyed.

**Table 1.** **Model outputs for the associations a Gaston rare species had with species diversity, evenness and density.**
Negative binomial generalised linear model outputs tested for the associations between vegetation community composition variables (species diversity, evenness and density) with the number of rare plants at a site, classified by Gaston, across the 86 sites surveyed. Raw estimates, standardised regression coefficients and estimated 95% confidence intervals are shown. The reference level for vegetation community (Veg Type) and vegetation x vegetation community composition variable (against which other types are compared) is forest.

|  | **Species diversity** | | | **Species evenness** | | | **Species density** | | |
| --- | --- | --- | --- | --- | --- | --- | --- | --- | --- |
| *Coefficient* | *Log-Mean* | *Conf. Int (95%)* | *P-Value* | *Log-Mean* | *Conf. Int (95%)* | *P-Value* | *Log-Mean* | *Conf. Int (95%)* | *P-Value* |
| Intercept | 0.81 | 0.04 – 1.58 | **0.040** | 1.97 | -1.37 – 5.30 | 0.248 | 1.15 | 0.63 – 1.68 | **<0.001** |
| Veg Type [heath] | 0.24 | -0.92 – 1.39 | 0.688 | 4.36 | -0.63 – 9.35 | 0.087 | 0.87 | -0.12 – 1.86 | 0.086 |
| Veg Type [woodland] | 1.10 | 0.04 – 2.17 | **0.043** | -7.20 | -14.09 – -0.31 | **0.040** | 0.06 | -0.85 – 0.96 | 0.901 |
| Alpha diversity | 0.05 | 0.00 – 0.10 | **0.035** |  |  |  |  |  |  |
| Elevation | -0.00 | -0.00 – 0.00 | 0.588 | 0.00 | -0.00 – 0.01 | 0.319 | -0.00 | -0.00 – 0.00 | 0.826 |
| Aspect-Eastings | 0.02 | -0.15 – 0.19 | 0.782 | 0.11 | -0.06 – 0.27 | 0.198 | 0.09 | -0.08 – 0.26 | 0.296 |
| Aspect-Northings | -0.06 | -0.29 – 0.16 | 0.587 | -0.06 | -0.28 – 0.15 | 0.566 | -0.04 | -0.25 – 0.17 | 0.707 |
| Veg Type [heath]*Alpha diversity | -0.01 | -0.08 – 0.07 | 0.844 |  |  |  |  |  |  |
| Veg Type [woodland]*Alpha diversity | -0.03 | -0.09 – 0.03 | 0.262 |  |  |  |  |  |  |
| Species evenness |  |  |  | -0.57 | -4.78 – 3.64 | 0.791 |  |  |  |
| Veg Type [heath]*Species evenness |  |  |  | -5.64 | -12.03 – 0.75 | 0.084 |  |  |  |
| Veg Type [woodland]*Species evenness |  |  |  | 9.72 | 1.22 – 18.22 | **0.025** |  |  |  |
| Species density |  |  |  |  |  |  | 0.00 | -0.00 – 0.00 | 0.068 |
| Veg Type [heath]*Species density |  |  |  |  |  |  | -0.00 | -0.00 – 0.00 | 0.088 |
| Veg Type [woodland]*Species density |  |  |  |  |  |  | 0.00 | -0.00 – 0.00 | 0.231 |
| Observations | 86 | | | 86 | | | 86 | | |
| R^2^ | 0.543 | | | 0.582 | | | 0.595 | | |

**Table 2.** **Model outputs for the associations a Rabinowitz rare species had with species diversity, evenness and density.**Negative binomial generalised linear model outputs tested for the associations between vegetation community composition variables (species diversity, evenness and density) with the number of rare plants at a site, classified by Rabinowitz, across the 86 sites surveyed. Raw estimates, standardised regression coefficients and estimated 95% confidence intervals are shown. The reference level for vegetation community (Veg Type) and vegetation x vegetation community composition variable (against which other types are compared) is forest.

|  | **Species diversity** | | | **Species evenness** | | | **Species density** | | |
| --- | --- | --- | --- | --- | --- | --- | --- | --- | --- |
| *Coefficient* | *Log-Mean* | *Conf. Int (95%)* | *P-Value* | *Log-Mean* | *Conf. Int (95%)* | *P-Value* | *Log-Mean* | *Conf. Int (95%)* | *P-Value* |
| Intercept | 0.92 | 0.35 – 1.50 | **0.002** | -0.41 | -3.36 – 2.54 | 0.785 | 1.50 | 1.11 – 1.89 | **<0.001** |
| Veg Type [heath] | 0.68 | -0.15 – 1.51 | 0.110 | 0.26 | -4.12 – 4.64 | 0.907 | -0.01 | -0.76 – 0.74 | 0.982 |
| Veg Type [woodland] | 0.87 | -0.08 – 1.82 | 0.072 | -0.03 | -7.95 – 7.89 | 0.994 | -0.57 | -1.49 – 0.35 | 0.224 |
| Alpha diversity | 0.09 | 0.05 – 0.12 | **<0.001** |  |  |  |  |  |  |
| Elevation | -0.01 | -0.01 – -0.00 | **<0.001** | -0.00 | -0.01 – -0.00 | **0.024** | -0.00 | -0.01 – -0.00 | **0.002** |
| Aspect-Eastings | -0.14 | -0.28 – -0.01 | **0.040** | -0.09 | -0.25 – 0.06 | 0.232 | -0.16 | -0.31 – -0.02 | **0.027** |
| Aspect-Northings | -0.15 | -0.34 – 0.04 | 0.131 | -0.14 | -0.36 – 0.08 | 0.200 | -0.12 | -0.31 – 0.07 | 0.202 |
| Veg Type [heath]*Alpha diversity | -0.02 | -0.08 – 0.04 | 0.490 |  |  |  |  |  |  |
| Veg Type [woodland]*Alpha diversity | -0.08 | -0.13 – -0.03 | **0.001** |  |  |  |  |  |  |
| Species evenness |  |  |  | 3.27 | -0.45 – 6.98 | 0.085 |  |  |  |
| Veg Type [heath]*Species evenness |  |  |  | 0.14 | -5.44 – 5.71 | 0.962 |  |  |  |
| Veg Type [woodland]*Species evenness |  |  |  | -0.39 | -10.16 – 9.37 | 0.937 |  |  |  |
| Species density |  |  |  |  |  |  | 0.00 | 0.00 – 0.00 | **<0.001** |
| Veg Type [heath]*Species density |  |  |  |  |  |  | 0.00 | -0.00 – 0.00 | 0.657 |
| Veg Type [woodland]*Species density |  |  |  |  |  |  | 0.00 | -0.00 – 0.00 | 0.847 |
| Observations | 86 | | | 86 | | | 86 | | |
| R^2^ | 0.611 | | | 0.390 | | | 0.610 | | |
